# Oxidation of interfacial cobalt controls the pH dependence of the oxygen evolution reaction

**DOI:** 10.1038/s41557-025-01784-1

**Published:** 2025-03-28

**Authors:** Jinzhen Huang, Adam H. Clark, Natasha Hales, Kenneth Crossley, Julie Guehl, Radim Skoupy, Thomas J. Schmidt, Emiliana Fabbri

**Affiliations:** 1 PSI Center for Energy and Environmental Sciences, Paul Scherrer Institute, Villigen, Switzerland; 2 PSI Center for Photon Sciences, Paul Scherrer Institute, Villigen, Switzerland; 3 PSI Center for Life Sciences, Paul Scherrer Institute, Villigen, Switzerland; 4 Institute for Physical Molecular Sciences, ETH Zürich, Zürich, Switzerland

**Keywords:** Electrocatalysis, Energy

## Abstract

Transition metal oxides often undergo dynamic surface reconstruction under oxygen evolution reaction conditions to form the active state, which differs in response to the electrolyte pH. The resulting pH dependency of catalytic activity is commonly observed but poorly understood. Herein we track Co oxidation state changes at different pH-directed (hydr)oxide/electrolyte interfaces using operando X-ray absorption spectroscopy characterizations. Combined with in situ electrochemical analyses, we establish correlations between Co redox dynamics, the flat band potential and Co oxidation state changes to explain the pH dependency of the oxygen evolution activity. Alkaline environments provide a low flat band potential that yields a low-potential Co redox transformation, which favours surface reconstruction. Neutral and acidic environments afford an anodic shift of the Co redox transformation that increases the catalytic overpotential. The larger overpotential in neutral environments is attributable to poor Co atom polarizability and slow Co oxidation state changes. These findings reveal that interfacial Co oxidation state changes directly determine the pH dependency of the oxygen evolution reaction activity.

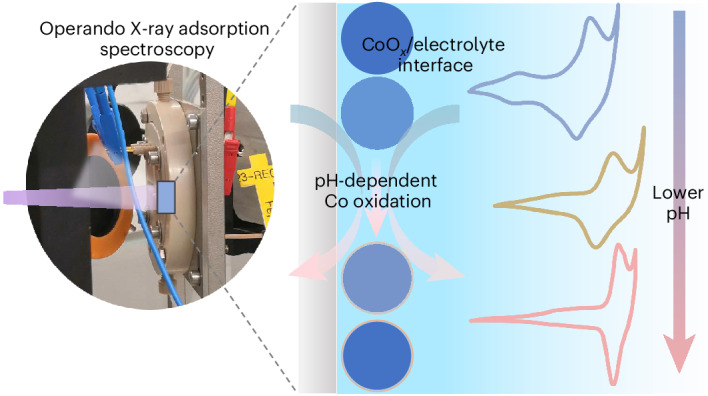

## Abstract (web summary)

Transition metal oxides serve as important electrocatalysts for the oxygen evolution reaction, although Co-based oxides typically undergo pH-dependent dynamic surface reconstruction under catalytic conditions. Now the correlations between Co redox dynamics, flat band potential and Co oxidation state changes have been established to explain the pH dependency of oxygen evolution activity.

## Main

The physicochemical properties of a catalyst/electrolyte interface determine the characteristics of a heterogeneous electrocatalytic reaction^1^. Such properties include the pristine catalyst surface structure, its interfacial structure under operando conditions^2^, the interfacial H^+^/OH^−^ bond network^3,4^ and the nature of the electrolyte cations^5–7^ and anions^8,9^. The situation is more complicated if surface reconstruction is coupled with the catalytic reaction. Taking the oxygen evolution reaction (OER) as an example, the applied oxidation potentials facilitate the reconstruction of the catalyst surface^10–13^, in response to the electrolyte pH, to determine the ultimate electrocatalytic performance.

The OER plays an important role in many sustainable technologies, including solar/electrochemical water splitting, metal–air batteries, CO_2_ reduction, N_2_ fixation and so on^14–16^. The four-electron OER process is kinetically slow and thermodynamically unfavourable; hence it must be driven by high overpotentials, which results in high energy consumption^17^. Tremendous efforts have been devoted to the development of efficient and cost-effective OER catalysts^13,18,19^. The metallic-conductive noble metal oxides (for example, RuO_2_ and IrO_2_) display excellent OER activity in all pH environments^20–23^, with very limited surface reconstruction^24–26^. Their OER activity is generally higher in acidic than in alkaline environments^20,21^. Conversely, catalysts based on comparatively abundant cobalt oxides are semiconducting^27^ and undergo significant surface reconstruction^12,28,29^, generally presenting higher OER performance in alkaline^30,31^, rather than acidic, environments^28,29,32,33^. More importantly, the OER activity of cobalt-based oxides is far lower than that of IrO_2_/RuO_2_ under acidic conditions. These differences underscore that the reconstructed cobalt (hydr)oxide/electrolyte interface plays an important role in determining the pH dependence of OER activity.

Currently, research has suggested that OER activity is dependent on the cations^34,35^ and anions^8,36,37^ present in the electrolyte, and on the pH value^20,21,23,38^. Under these circumstances, applying the well-established activity descriptors based on intermediate adsorption energies^17,39^ and the related Sabatier principle^14^ to predict the OER process of a catalyst is difficult. While the adsorption energies of OER intermediates can be pH dependent and still follow the scaling relation, research has proposed that the Sabatier principle is insufficient to explain the pH dependency of OER activity^20,21^, possibly due to the catalyst surface reconstruction. In addition, research suggests the OER current is controlled by the charge accumulated at the interface, not directly by the applied potential^24^, highlighting the important contribution of surface charge to the reaction process. Therefore, the pH dependency of OER activity should manifest from the dual effects of both surface reconstruction and charge accumulation at pH-directed interfaces, which are all related to the oxidation of the metal centre^24,40^. Previously, we demonstrated that time-resolved operando hard X-ray absorption spectroscopy (hXAS) characterizations^41–43^ at the Co K edge can track the dynamic Co oxidation state changes under OER conditions^12,27,44,45^. Monitoring the operando Co oxidation state changes at different pH-directed interfaces is a straightforward method to probe the surface reconstruction and charge accumulation, thus uncovering the mechanism that controls the pH dependency of OER activity.

**Fig. 1:**
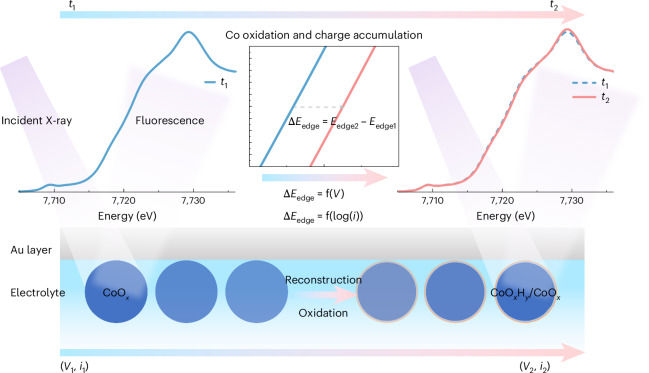
Tracking the Co oxidation state changes by operando hXAS characterization. Fast data acquisition enables monitoring of the Co K-edge energy shift (Δ*E*_edge_) of CoO_*x*_ under operando conditions as a function of the applied potential (Δ*E*_edge_ = f(*V*) or logarithm of current (Δ*E*_edge_ = f(log(*i*)). Source data

Herein we apply operando hXAS to track the Co oxidation state of commercial nano-size CoO_*x*_ at different applied potentials and in different electrolytes. The change in Co oxidation state under operando conditions is captured by the energy shift of the Co K edge (Δ*E*_edge_)^44^ between the initial state at time *t*_1_(*V*_1_, *i*_1_) and another state at the operating time *t*_2_(*V*_2_, *i*_2_), as a function of the applied potential (*V*) or logarithm of current (*i*; Fig. 1). The Δ*E*_edge_ in response to the surface Co oxidation state changes reflects both irreversible surface reconstruction to form an oxidized layer, and reversible OER-dynamic charging/discharging in response to the applied potential. The onset of Δ*E*_edge_ reveals that the Co oxidation state changes correlate with the formal potentials (*V*°) of the Co^II/III^ redox process and the flat band potential. The formation of Co^IV^ species is the potential determining step for the OER^46^ in all tested electrolytes. The anodic shift of Co^III/IV^ redox with decreasing electrolyte pH contributes significantly to the higher overpotential in neutral and acidic environments. Among the pH regimes, the neutral environment has the least favourable surface reconstruction before the Co^III/IV^ redox, and the slowest Co oxidation state changes with respect to the change in applied potential (or OER current) under OER conditions, resulting in the largest OER overpotential. These findings elucidate the connections between Co redox dynamics, the Co oxidation state changes and the pH dependency of OER activity at different interfaces.

## Results and discussion

### Structural characterizations of nano-size CoO_*x*_

Nano-sized CoO_*x*_ is composed primarily of spinel Co_3_O_4_ with a minor rocksalt CoO component (Supplementary Figs. 1 and 2). Ex situ hXAS and soft X-ray absorption spectroscopy (sXAS) characterizations were performed to compare the bulk and surface oxidation states of nano CoO_*x*_ catalyst to those of commercial Co_3_O_4_ and CoO references. The bulk averaged Co oxidation state of nano CoO_*x*_ is around 2.52^+^, close to 2.67^+^ for the Co_3_O_4_ reference (Supplementary Fig. 3). The corresponding Fourier-transformed extended X-ray absorption fine-structure (EXAFS) spectrum of nano-sized CoO_*x*_ shows three representative scattering contributions of the spinel structure, with a Co–O of ~1.91 Å (Supplementary Table 1). Surface-sensitive Co L_3_ spectra show the coexistence of Co^III^ species and Co^II^ species (Supplementary Fig. 4). The existence of low-spin Co^III^ is essential to ensure good OER performance in acidic environments^45^. Accordingly, the branching ratios of Co L_3_ and Co L_2_ edges were calculated to be 0.58 (Supplementary Table 2), suggesting that Co is in the low-spin state^47,48^. The O K-edge spectra of nano-sized CoO_*x*_ is dominated by the low-spin Co^III^ features in the region related to the Co 3*d* band^49^. To summarize, the electronic structure and surface state of nano-sized CoO_*x*_ are similar to those of the Co_3_O_4_ reference.

### The pH dependency of Co redox dynamics

**Fig. 2:**
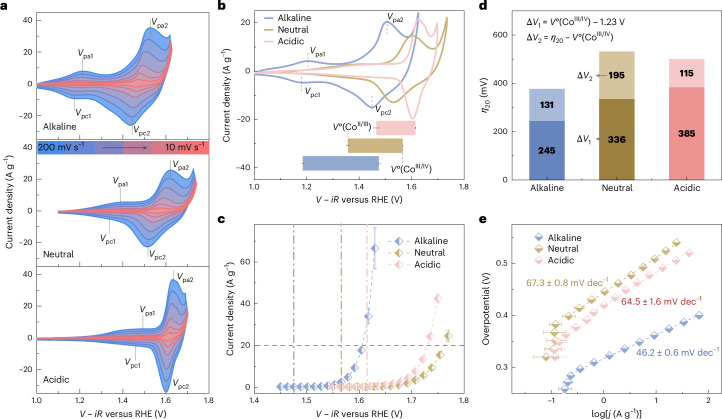
Co redox features and OER performance in different electrolytes. **a**, CV curves collected in alkaline, neutral and acidic electrolytes at scan rates from 200 to 10 mV s^−1^. *R* is the electrolyte resistance (ohms) in the rotating disc electrode (RDE) cell used for the correction to obtain *V* − *iR*. **b**, CV curves showing the Co^II/III^ (*V*_pa1_ and *V*_pc1_) and Co^III/IV^ (*V*_pa2_ and *V*_pc2_) redox features in different electrolytes at a scan rate of 100 mV s^−1^. The inset bar chart shows the formal potentials (*V*°) of Co^II/III^ and Co^III/IV^ redox couples in different electrolytes. The standard deviation (s.d.) is obtained from averaging data points (*n* ≥ 5) collected at different scan rates, as detailed in Supplementary Fig. 6 and Supplementary Table 3. Data are presented as mean values ± s.d. **c**, The OER polarization curves collected by steady-state chronoamperometry at different constant applied potentials. The vertical dashed lines indicate the *V*°(Co^III/IV^) in different electrolytes. **d**, The stacked bar chart summarizes the *η*_20_, Δ*V*_1_ and Δ*V*_2_ values. **e**, The Tafel plot derived from the OER polarization curves. The s.d. in **c** and **e** is from averaging three individual measurements. Data are presented as mean values ± s.d. Source data

Cyclic voltammetry (CV) measurements conducted at different scan rates showcase the Co redox dynamics at different interfaces (Fig. 2a and Supplementary Figs. 5 and 6). The two pairs of redox peaks (anodic and cathodic) appearing from low to high applied potential are assigned to Co^II/III^ (*V*_pa1_ and *V*_pc1_) and Co^III/IV^ (*V*_pa2_ and *V*_pc2_) transitions^28,46,50^. Charge transferred during these two redox processes is reflected in the integrated CV area, which is approximately ×1.5 greater in the alkaline electrolyte than in the acidic and neutral electrolytes, indicating the redox-active Co site is more abundant in the alkaline electrolyte (Supplementary Fig. 7). The formal redox potentials (*V*° = (*V*_pa_ + *V*_pc_)/2, in which *V*_pa_ and *V*_pc_ are the potentials of the anodic and cathodic redox peaks, respectively) of the two Co redox couples are given in Fig. 2b, with details in Supplementary Fig. 6 and Supplementary Table 3. Since both proton and electron transfer are involved in the Co redox processes, the anodic shift with decreasing pH of both Co redox couples on the reversible hydrogen electrode (RHE) scale indicates that the deprotonation/protonation is affected by the pH environment. These super-Nernstian shifts suggest the transferred number of H^+^/OH^−^ is not equal to the number of electrons (*e*) during redox processes^51–53^. In addition, the super-Nernstian shifts of Co^II/III^ redox and Co^III/IV^ redox potentials^28,52,54^ are different, as confirmed by a smaller potential difference between the *V*° of Co^II/III^ and Co^III/IV^ redox couples in a lower pH environment (bar chart in inset of Fig. 2b). Concomitantly, the separation between the *V*_pa2_ and *V*_pc2_ of Co^III/IV^ redox is the largest in a neutral electrolyte (Supplementary Fig. 8), indicating that Co^III/IV^ redox is electrochemically less reversible due to the slow interfacial electron transfer^55^.

Alongside electron transfer, H^+^/OH^−^ transport at the interface also influences the redox dynamics. The dependence of the redox peak intensity (*j*) on the scan rate (*v*) follows the power-law relationship (*j* = *av*^*b*^, where *a* and *b* are fitting constants; Supplementary Fig. 9). The extracted *b* value for the first Co^II/III^ redox couple is closer to 1 than the *b* value for the Co^III/IV^ redox couple, indicating the latter process is more affected by H^+^/OH^−^ diffusion^56,57^. The *b* value for *V*_pc2_ increases in low pH environments, to a value that is comparable to that of Co^II/III^ redox peaks in the acidic electrolyte. Therefore, Co redox dynamics are not only related to the original cobalt oxide structure and surface, but also significantly affected by the (hydr)oxide/electrolyte interface that forms in situ during electrocatalysis.

### The pH dependency of OER activity

Chronoamperometry measurements were performed at different potentials to obtain OER polarization curves in different electrolytes (Fig. 2c). The overpotentials at a mass current density of 20 A g^−1^ (*η*_20_) are 376, 531 and 500 mV in alkaline, neutral and acidic electrolytes, respectively. Generally, the Co^III/IV^ redox process is important for the formation of active phases and the initiation of the OER for Co-based catalysts^50,58^. However, the Co^III/IV^ formal redox potential increases in a low pH environment (Fig. 2b). Using the parameter Δ*V*_1_ = *V*°(Co^III/IV^) – 1.23 V we decouple the contribution of the pH-triggered anodic shift in the Co^III/IV^ redox process from the total *η*_20_. The potential difference Δ*V*_2_ = *η*_20_ – *V*°(Co^III/IV^) increases from 115 to 131 to 195 mV in acidic, alkaline and neutral electrolytes, respectively (Fig. 2d). Therefore, the thermodynamically unfavourable Co^III/IV^ redox transformation significantly contributes to the increased OER overpotential in an acidic environment; in a neutral environment, the largest Δ*V*_2_ suggests that an additional parameter or parameters hinder the OER process. Tafel plots were derived from OER polarization curves (Fig. 2e) to show that Tafel slopes increase from 46.2 ± 0.6 to 64.5 ± 1.6 to 67.3 ± 0.8 mV dec^−1^ in alkaline, acidic and neutral electrolytes, respectively, demonstrating that the overall OER kinetics are optimal in an alkaline electrolyte. The observed pH dependency of OER activity can be related to the different Co oxidation state changes at different interfaces, which will be further discussed in combination with the operando hXAS results in Fig. 5.

### Electrochemistry of semiconductors: the flat band potential

**Fig. 3:**
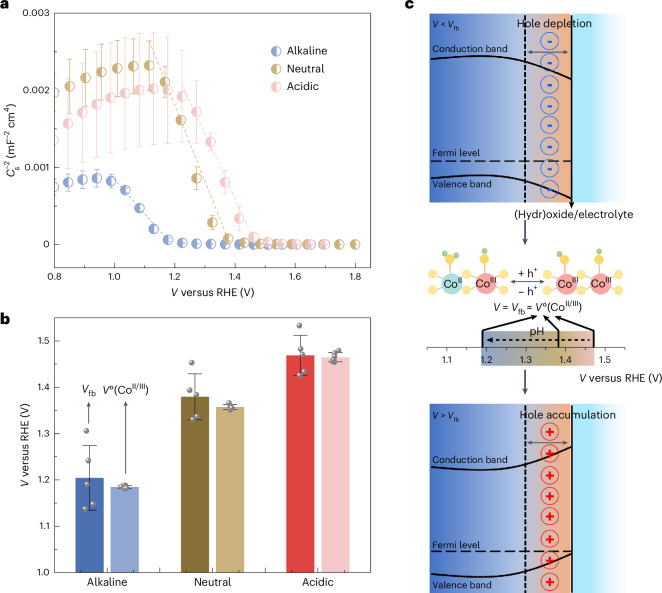
Coupling of Co redox process with the flat band potential and interfacial charge accumulation. **a**, Mott–Schottky plots derived using a frequency of 1,000 Hz; the s.d. is obtained from averaging three individual data points of repetition. Data are presented as mean values ± s.d. The Mott–Schottky plots derived from other frequencies are shown in Supplementary Fig. 11. **b**, Comparison of the flat band potential (*V*_fb_) and *V*°(Co^II/III^). The s.d. of *V*_fb_ bars are obtained by averaging of the data points at different frequencies (*n* = 5). The sample size to obtain the s.d. of *V*°(Co^II/III^) is detailed in Supplementary Fig. 6. Data are presented as mean values ± s.d. **c**, Schematic demonstration on the coupling between the Co^II/III^ redox process and shift of interfacial properties at the (hydr)oxide/electrolyte interface. h^+^, hole. Source data

In situ electrochemical impedance spectroscopy was performed to understand how the (hydr)oxide/electrolyte interfaces respond to applied potential (Supplementary Fig. 10). The space charge capacitance (*C*_s_) of semiconductor/electrolyte interfaces is related to the charge accumulation and is frequency dispersive^59^ (details in Supplementary Note I and Supplementary Figs. 11 and 12). The ${{{C}}}_{{\rm{s}}}^{-2}$ value extracted at different frequencies decreases with potential to give a negative Mott–Schottky slope for all interfaces, indicating that the surface-reconstructed catalyst behaves as a p-type semiconductor^60,61^ (Fig. 3a). The apparent flat band potential (*V*_fb_) and the number of interfacial holes (*N*_h_, inversely proportional to the Mott–Schottky slope)^60,61^ both vary slightly with frequency (Supplementary Fig. 11). The trend in the Mott–Schottky slope is alkaline < acidic < neutral; inversely, the abundance of active sites at different interfaces follows the order of alkaline > acidic > neutral. The *V*_fb_ increases in lower pH environments, and the average *V*_fb_ extracted for different interfaces matches well with the corresponding *V*°(Co^II/III^), indicating the change in interfacial properties is significantly coupled with the Co redox process (Fig. 3b). This result is consistent with apparent band-edge energies for semiconductors being affected by the charge accumulation and shifting with pH, electrolyte type and surface chemistry^62^. As the catalyst surface undergoes a transition from hole depletion to hole accumulation at potentials above *V*_fb_ (Fig. 3c), the holes injected from the electrolyte can be stored at the catalyst surface via the oxidation of Co centres or the formation of oxygen ligand holes^50,63^. The Co^II^ species accept the holes (that is, by losing an electron) to form Co^III^ species during the oxidation process, associated with the surface reconstruction. The interfacial capacitances extracted by a CV method increase by around one order of magnitude in all the electrolytes after the *V*_fb_ (details in Supplementary Figs. 13 and 14). These observations verify the strong coupling among the redox process, charge accumulation, capacitance and apparent *V*_fb_ at different interfaces. Furthermore, the Co^II/III^ redox process in acidic conditions is delayed by ~300 mV compared to alkaline conditions, suggesting that interfacial charge accumulation via oxidization of Co active centres is much more thermodynamically unfavourable. The highest concentration of active sites and the lowest potential to start charge accumulation explains the superior OER activity of CoO_*x*_ in an alkaline electrolyte.

### Operando hXAS characterizations

**Fig. 4:**
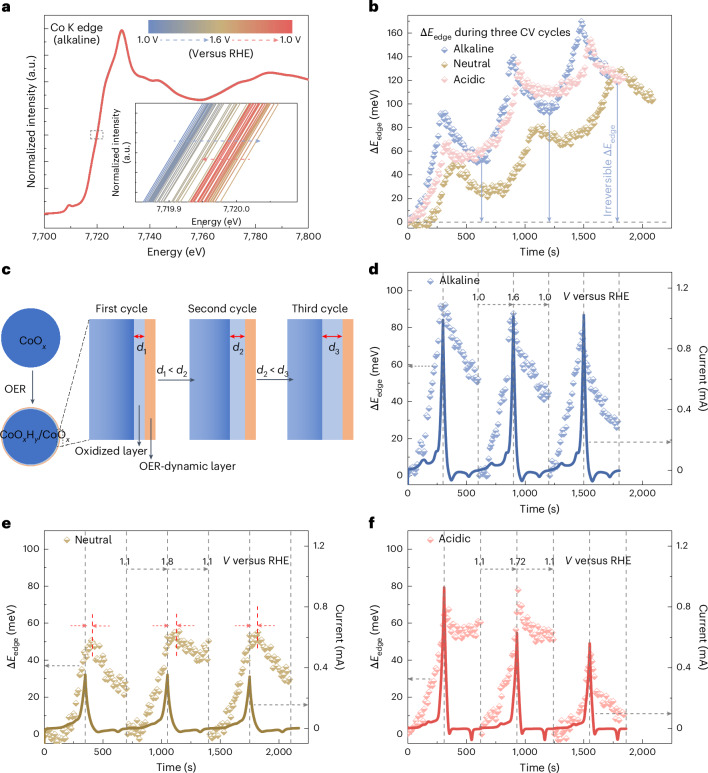
Operando hXAS characterization during CV measurement. **a**, The operando X-ray absorption near-edge structure spectra at the Co K edge during the first CV measurement in alkaline electrolyte. The inset shows a zoomed-in view with the changes of *E*_edge_. **b**, The Δ*E*_edge_ (calculated by taking the *E*_edge_ at the start of the first CV measurement as the initial state) observed during the CV measurement in different electrolytes. **c**, Schematic demonstration of the formation of reconstructed layers (including the oxidized layer and OER-dynamic layer) at the interface. *d*_1_, *d*_2_ and *d*_3_ denote the increasing thickness of the oxidized layer with cycling. **d**–**f**, The Δ*E*_edge_ (calculated by taking the *E*_edge_ at the start of each CV measurement as the initial state) observed during each CV cycle in alkaline (**d**), neutral (**e**) and acidic (**f**) environments. The red arrows and dashed lines in (**e**) indicate the mismatch between the peaks of *ΔE*_edge_ and peaks of OER current in the neutral environment. Source data

To study the interfacial Co oxidation state changes, we carried out operando hXAS characterizations at the Co K edge during CV measurements (Supplementary Figs. 15 and 16; the procedure is detailed in Supplementary Note II and the Methods). The Co oxidation state change during CV measurement in an alkaline environment is tracked by the shift at the Co K edge (Fig. 4a). The *E*_edge_ of the Co K edge increases with applied potential; however, this change is not fully reversible on the reverse cathodic scan. Similar irreversible changes in the Co oxidation state also happen in neutral and acidic electrolytes (Supplementary Fig. 17). The *E*_edge_ was extracted by an integral method^28,51^, and the Δ*E*_edge_ (calculated by setting the *E*_edge_ at the start of the first CV measurement as the initial state) during three CV cycles is demonstrated in Fig. 4b. The irreversible Δ*E*_edge_ increases with CV cycles in all pH environments, indicating the reconstructed (hydr)oxide layers become thicker during the operating time (Fig. 4c), as also suggested in the literature^64,65^. The reconstruction process is clearly pH dependent, since the Δ*E*_edge_ increases to 94 meV and decreases to 52 meV in the first cycle in the alkaline environment, while the corresponding extrema are 50 and 28 meV in neutral electrolyte, and 65 and 60 meV in acidic electrolyte. The irreversible Δ*E*_edge_ from the thickening of (hydr)oxide layers is comparable to the potential-driven Δ*E*_edge_ in each electrolyte (that is, the Δ*E*_edge_ observed before the onset of OER; details in Supplementary Fig. 18). These irreversible changes under an open circuit potential after the CV measurement are also dependent on the electrolytes (Supplementary Figs. 19 and 20). The bulk-sensitive EXAFS spectra reveal the initial Co_3_O_4_-like structure was maintained after the OER in all electrolytes (Supplementary Fig. 21 and Supplementary Table 4). The observed changes in the Co K edge arise primarily from the reconstructed CoO_*x*_H_*y*_ surface.

Moreover, some Co sites at the interface are oxidized/reduced with an obvious periodic change in response to the anodic/cathodic scans, respectively. Therefore, the reconstructed surface can be described in two parts, namely the oxidized layer that thickens over CV cycling and the surface OER-dynamic layer that reversibly oxidizes/reduces when the applied potential is increased/decreased (Fig. 4c). The oxidized layer thickens because some of the oxidized Co^III^ species cannot recover to the initial state upon contact with the electrolyte; while the periodic change of Δ*E*_edge_ due to the change of the OER-dynamic layer is contributed to by the formation of both the real OER-active Co species and other spectator Co species simultaneously formed under OER conditions (Supplementary Note III for more details). To better demonstrate how the changes in Co oxidation state of the OER-dynamic layer correlate with the OER performance, the Δ*E*_edge_ at each CV cycle is calculated with respect to the start of each CV (scatter profiles in Fig. 4d–f). The Δ*E*_edge_ (and therefore the change of Co oxidation state) is closely related to the OER current (line profiles in Fig. 4d–f) in each electrolyte. The OER current is almost unchanged and therefore the magnitude of changes in Δ*E*_edge_ is stable with cycling in alkaline and neutral environments. In an acidic electrolyte, the maximum OER current decreases with cycling, possibly due to Co dissolution, before forming a quasi-stable interface, resulting in a lower Δ*E*_edge_ at the Co K edge in the third CV cycle. The mismatch between the maximum OER current and maximum Δ*E*_edge_ seen in the neutral environment (Fig. 4e) can be explained by continued Co oxidation at the beginning of the cathodic scan driven by high applied potential. In addition, the measured potential is slightly affected by a change of the local pH environment in neutral electrolyte (Supplementary Note IV for more details). In general, the dynamic changes in Δ*E*_edge_ over CV cycling show that the Co oxidation state changes are sensitive to the applied potential (or OER current) at different (hydr)oxide/electrolyte interfaces.

### The pH dependency of Co oxidation state changes

**Fig. 5:**
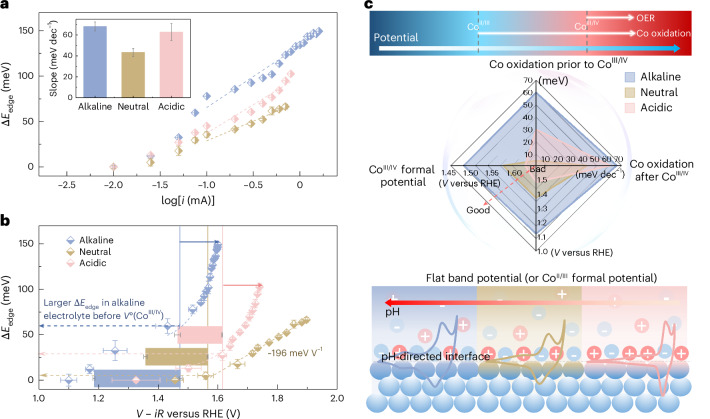
Operando hXAS characterization during CP measurement. **a**,**b**, The Δ*E*_edge_ of the Co K edge, collected during CP measurement in a flow cell, is plotted as a function of log(*i*) (**a**) and applied potential (**b**). The measured resistance *R* in the flow cell is used for the correction to obtain *V* − *iR* in (**b**). The s.d. of Δ*E*_edge_ is obtained from averaging three data points. The s.d. of applied potentials is obtained from averaging all the potentials recorded at each step (*n* = 120), as detailed in Supplementary Fig. 22. Data are presented as mean values ± s.d. The inset in **a** shows the slopes with the error obtained from a linear fitting. The formal redox potentials inset in **b** are adapted from Fig. 2b. **c**, Summary of different parameters that contribute to the pH dependency of OER activity. The top diagram shows the oxidation of interfacial Co starts after the Co^II/III^ redox while the OER starts after the Co^III/IV^ redox. The middle plot summarizes the pH-dependent Co redox potentials and Co oxidation. The bottom scheme demonstrates the Co redox processes vary at pH-directed interfaces. Source data

To obtain further insight into the correlations between the interfacial Co oxidation state changes and OER activity, a steady-state chronopotentiometry (CP) technique was applied during the operando hXAS characterizations (Supplementary Figs. 22 and 23). At a low controlled current, positive charging of the interface and oxidation of the Co sites are the dominant processes, rather than the OER. The Δ*E*_edge_ (calculated by setting the *E*_edge_ at 0.01 mA as the initial state) shows a quasi-linear relationship with log(*i*) (Fig. 5a), indicating the OER current is controlled by the Co oxidation state at the interface, similar to how the accumulated oxidation charge affects the OER current in IrO_*x*_ catalysts^24^. Furthermore, the linear fit of the slope (at *i* ≥ 0.1 mA) yields the change in Δ*E*_edge_ when the OER current increases by one order of magnitude (inset in Fig. 5a), which is comparable in alkaline and acidic electrolytes but smaller in the neutral electrolyte. Therefore, the formation of active phases with respect to the change in applied potential (or OER current) is the slowest in the neutral environment, affecting the number of active sites.

As discussed previously, the Co oxidation state changes indicated by Δ*E*_edge_ are a convolution of oxidizing the surface-active layer and thickening the subsurface layer. The Δ*E*_edge_ results suggest that when the surface reconstruction is coupled with the OER, the accumulated oxidation charges in both the reconstructed subsurface layer (that is, spectator Co species) and the OER-active Co species are still linearly correlated to the OER current (Fig. 5a). Similarly, a good linear correlation is still observed from the log–log plot of OER current versus total charge when several types of holes are at the interface of a haematite photoanode^66^. For the CoO_*x*_ studied here, the total charge extracted by the pulse voltammogram analysis^24^ is also linearly correlated with the logarithm of OER current in different electrolytes (Supplementary Fig. 24). These results indicate the important role of pH-directed interfaces in determining the Co oxidation state changes and charge accumulation. Besides, the Co oxidation state changes haven been proven to be influenced by the nanoparticle size^63^. We prove that the Co_3_O_4_ control sample with a smaller specific area than the nano-sized CoO_*x*_ shows a similar irreversible Δ*E*_edge_ due to the formation of an (oxy)hydroxide layer (Supplementary Figs. 25–28). Even the overall Co oxidation is affected by the specific surface area of the catalyst; log(*i*) and Δ*E*_edge_ are still in a good linear relationship, supporting the conclusion that formation of different types of oxidized species is strongly coupled with the OER (Supplementary Note III for details).

The Δ*E*_edge_ is also plotted as a function of the applied potential and compared with the Co redox formal potentials (Fig. 5b). From this perspective, three important points can be extracted from the plots. First, the Δ*E*_edge_ starts to increase close to the corresponding *V*°(Co^II/III^) in all the interfaces, confirming the direct correlation between the Co redox process and formation of the oxyhydroxide layer. Second, the Δ*E*_edge_ observed prior to the Co^III/IV^ formal potentials (vertical lines in Fig. 5b) is the most significant in an alkaline electrolyte (~60 meV), medium in acidic electrolyte (~30 meV) and a minimum in neutral electrolyte (~5 meV), consistent with the smallest potential-driven Co oxidation state changes in neutral electrolyte (Supplementary Fig. 18). Third, following the Co^III/IV^ formal potentials, the Δ*E*_edge_ in alkaline and acidic electrolytes grows pseudo-exponentially, with a comparable increase of ~80 meV in Δ*E*_edge_ when the applied potential increases by 0.12 V, which explains the more comparable Δ*V*_2_ (131 versus 115 mV) extracted in Fig. 2d. By comparison, the Δ*E*_edge_ in the neutral electrolyte still increases slowly, with a linear slope of ~196 meV V^−1^ after the Co^III/IV^ redox transformation. The slowest accumulation of oxidized Co species following Co^III/IV^ redox, which is a function of OER current (Fig. 5a) or applied potential (Fig. 5b), represents an important parameter to consider in explaining the lowest OER activity and the largest Δ*V*_2_ (195 mV in Fig. 2d) in a neutral environment.

The pH dependency of the electrified interfaces is revealed by differences in the flat band potential and formal potential of the redox couples, which in turns affects the Co oxidation state changes and the OER activity. These are identified in the present study as key descriptors of the OER pH dependency for Co-based catalysts and summarized in Fig. 5c. The reconstructed interfaces show p-type semiconducting properties; therefore the flat band potential, related to the *V*°(Co^II/III^), controls the interfacial charge accumulation^67^. The super-Nernstian shift of the Co^II/III^ redox process^54^ is accompanied by the increased flat band potential in low pH environments. Likewise, the *V*°(Co^III/IV^) also shifts positively at low pH (Fig. 2b), indicating that the formation of OER-active Co^IV^ species^46,50,58^ is thermodynamically less favourable in neutral and acidic electrolytes. Therefore, we note that the thermodynamic shifts in the Co redox resulting from the semiconductor properties, and the unequal proton–electron transfer during redox processes at the interface are the important descriptors for the pH dependency of OER activity.

The Co oxidation state changes, indicated by Δ*E*_edge_, can be driven by the potential before the *V*°(Co^III/IV^) and coupled with the OER after the *V*°(Co^III/IV^). The Δ*E*_edge_ prior to *V*°(Co^III/IV^) represents the pseudo-capacitive charge accumulated at the interface^40^ via oxidization of the Co centres, which is most significant in an alkaline environment but a minimum in a neutral environment. After the *V*°(Co^III/IV^), in the OER region, the quasi-linear relationship between Δ*E*_edge_ and log(*i*) demonstrates that the OER current is controlled by the Co oxidation state in Co oxides^51^, consistent with the charge–current relationship found in IrO_*x*_ (ref. ^24^). However, the Δ*E*_edge_ varies in different interfaces, elucidating the pH dependence of Co oxidation state changes. Specifically, from the perspectives of both OER current and applied potential, Co oxidation state changes in alkaline and acidic electrolytes are comparably fast, but slowest in a neutral environment. Therefore, the high overpotential in acidic electrolytes is primarily due to the thermodynamic upshift of *V*°(Co^III/IV^), delaying the formation of Co^IV^ species at the interface. In the neutral environment, the surface reconstruction is the least favourable prior to Co^III/IV^ redox; after the Co^III/IV^ redox, the Co oxidation state changes are also the slowest among the pH regimes with respect to the change in applied potential (or OER current). Therefore, the low interfacial polarizability associated with Co oxidation contributes significantly to the highest overpotential in the neutral electrolyte. Recently, Liang et al. showed that the active-site coverage and the absorbate–absorbate interactions in IrO_*x*_ play important roles in controlling the OER process^68^. In particular, the repulsive interactions between active sites is pH dependent and stronger in alkaline electrolytes^23^. In CoO_*x*_, the coverage of active sites is related to in situ interfacial reconstruction, which is also pH dependent. Therefore, isolating the effects of pH-dependent active-site coverage and pH-dependent active-site interactions on the OER activity is difficult. However, the summary in Fig. 5c suggests that the pH-dependent active-site coverage plays a lead role in governing the OER activity of CoO_*x*_ in different electrolytes. Due to the similar chemical environments of the oxidized Co species, differentiating the OER-active species from other spectator Co species using the bulk-averaging hXAS characterization is difficult. Further investigation of the pH-dependent surface reconstruction using surface-sensitive sXAS^24^ at the Co L edge and O K edge could provide more details on the surface species at different interfaces.

### The pH dependency of Co dissolution

In addition to the activity, the stability of the Co^IV^ species at different (hydr)oxide/electrolyte interfaces was also investigated. First, the Co^IV^ to Co^III^ reduction peaks can be undoubtedly observed in all the investigated electrolytes from CV measurements (Fig. 1a). The open circuit potential curves recorded after holding the potential above the corresponding *V*°(Co^III/IV^) also reveal the plateaus assigned to Co^IV^ to Co^III^ reduction in all electrolytes (details in Supplementary Fig. 29), also due to the formation of Co^IV^ species. In addition, metal dissolution after the OER in different conditions has also been studied, to determine that Co^IV^ species are less stable in an acidic electrolyte (Supplementary Table 5). The stability number^69^ is calculated to be ~59 in acidic electrolyte, comparable to those of other Co-based catalysts (10^1^ to 10^2^) reported in the literature^70^. The surface-sensitive Co L-edge spectra (Supplementary Figs. 30 and 31) reveal that the Co species remain unchanged after the OER in alkaline electrolyte, which is consistent with the lack of obvious Co dissolution. This suggests the reconstructed CoO_*x*_H_*y*_ can reversibly change back to CoO_*x*_ after the OER (without contact with the electrolytes, under dry conditions). By comparison, the Co^III^ species are obviously reduced after the OER in acidic electrolyte. The Co^III^ species are oxidized into Co^IV^ species to catalyse the OER, but are partially dissolved into the electrolyte during the OER at low pH. This result supports our previous findings that low-spin Co^III^ is primarily responsible for the OER activity in an acidic environment^45^. In addition, Co dissolution is found to be affected by the electrochemical protocol and applied potential (discussed in Supplementary Note IV). Thus, finding an effective method to stabilize Co^III^/Co^IV^ species is key to solving the stability issue of Co-based OER catalysts in a low pH environment.

## Conclusions

In conclusion, combining in situ electrochemical analyses and operando XAS characterizations, we correlate the Co redox dynamics, surface reconstruction and OER activity of CoO_*x*_ under different pH values, that is, with different (hydr)oxide/electrolyte interfaces. The pH dependence of the OER activity is jointly controlled by the pH-induced thermodynamic shift of the Co redox transformation and the interfacial polarizability of Co atoms. The formal potentials for the Co^III/IV^ redox couples are higher in acidic than alkaline environments; however, the Co oxidation state changes following the Co^III/IV^ redox couple are comparable. Thus, the thermodynamic change of the Co redox process is mainly responsible for the higher OER overpotential in acidic environments. By comparison, the Co oxidation state changes in a neutral environment are thermodynamically unfavourable and the slowest with respect to the change in applied potential (or OER current). Under these circumstances, we elucidate that tuning the Co^III/IV^ redox behaviour to have a lower formal potential is key to reducing the overpotential for Co-based OER catalysts in an acidic environment. Additionally, engineering the catalyst/electrolyte interface to enhance polarizability, by changing the composition of the catalyst or the electrolyte, could facilitate charge accumulation in a neutral environment. These findings provide a new perspective to understand why the OER activity in different pH regimes disregards the Sabatier principle^20,21^ and guidance on designing efficient electrocatalysts appropriate for the pH environment.

## Methods

### Electrochemical characterizations in the rotating disc electrode set-up

All electrochemical data were collected using the Biologic ECLab software. The in situ electrochemical characterizations were conducted in a rotating disc electrode (RDE) set-up, with Au mesh as the counter electrode. Electrolytes composed of 0.1 M KOH (pH ~13), 0.1 M phosphate buffer (pH ~7) and 0.05 M H_2_SO_4_ solution (pH ~1) were used for the alkaline, neutral and acidic tests. A Hg/HgO reference electrode (filled with 0.1 M KOH) was used in 0.1 M KOH solution, while a Hg/HgSO_4_ reference electrode (filled with saturated K_2_SO_4_ solution) was used in 0.1 M phosphate buffer (prepared with 0.054 M:0.064 M of K_2_HPO_4_/KH_2_PO_4_) and 0.05 M H_2_SO_4_ solution. All potentials were reported against the RHE by calibrating the corresponding reference electrodes against a polycrystalline platinum disc, using the potential at zero current for hydrogen evolution/oxidation as 0 V versus RHE. To prepare the working electrode, nano CoO_*x*_ was mixed with Milli-Q water (18.2 Ω), 2-propanol and Nafion (Na^+^ exchanged) in a volume ratio of 200:50:1 to obtain an ink concentration of 2 g l^−1^. The as-prepared ink (~0.02 mg) was drop-cast on glassy carbon to obtain a thin layer of catalyst (~0.1 mg cm^−2^)^71^. The current measured in the RDE set-up (with a rotating speed of 1,600 rpm) was normalized by the mass of the catalyst. The working electrode was activated in the corresponding electrolyte by running CV for ten cycles to obtain a steady performance. Then, the OER polarization curves were obtained by performing chronoamperometry for 30 s and extracting the average current at each constant potential^27^.

### Mott–Schottky characterization

The Staircase Potentio Electrochemical Impedance Spectroscopy (Mott–Schottky) technique in the Biologic VMP-300 software was adapted for the Mott–Schottky characterization. The potential window was set to be 0.8 to 1.7 V versus RHE in alkaline and acidic electrolytes and 0.8 to 1.8 V versus RHE in neutral electrolyte, and the frequency was varied from 100 kHz to 1 Hz. The 1/*C*_s_^2^ parameter was extracted directly in the Biologic ECLab software at frequencies of 215, 464, 1,000, 2,154 and 4,641 Hz to derive the Mott–Schottky plots.

### Operando hXAS characterizations in the flow cell

The operando hXAS characterizations were performed at the SuperXAS beamline, Swiss Light Source, Paul Scherrer Institute. The incident beam from the 2.9 T superbend magnetic source was first collimated by a Si-coated mirror (2.9 mrad) and then was monochromatized by a Si(111) monochromator cooled with liquid nitrogen. The final beam size on the sample (1 × 0.4 mm^2^) was controlled by a Rh-coated double focusing mirror. The ion chambers were filled with 0.5 bar He or 0.5 bar N_2_. The data were collected in quick-scanning EXAFS mode^42^. The fluorescence spectra at the Co K edge were collected using a passivated implanted planar silicon detector^41^. The fast data acquisition enables the collection of two spectra per second. The Co K-edge spectra were collected at the energy range of 7,500 to 8,700 eV, using a metal Co foil as the reference. The operando electrochemical experiments were run in a flow cell developed in-house^72^. The working electrode was prepared by spraying the nano CoO_*x*_ ink with a catalyst concentration of 63.72 mg ml^−1^ in a H_2_O/Nafion mixture (volume ratio of 7/1) on an Au-coated (100 nm) Kapton foil to get a specific mass loading of ~2 mg cm^2^. This specific mass loading is higher than the ~0.1 mg cm^2^ of the RDE test. The catalyst layer is quite thick such that possibly not all the sprayed catalyst can access to electrolyte and catalyse OER. Therefore, the current collected in the flow cell is reported as collected but not normalized by the catalyst mass. The counter electrode was prepared by spraying carbon black on a similar Au-coated Kapton foil. A special Ag/AgCl reference electrode (2 mm in diameter and low electrolyte leakage; Harvard Apparatus) was used for the flow cell. The potential was calibrated to RHE by measuring the potential difference of the Ag/AgCl versus those used in the RDE set-up, in the corresponding electrolyte. CV and CP techniques were used during the operando hXAS measurement. The CV was run at an optimized potential window for CoO_*x*_ in different electrolytes for three cycles, at a scan rate of 2 mV s^−1^. The potential windows for alkaline, neutral and acidic electrolytes were optimized to be 1.0 to 1.6 V versus RHE, 1.1 to 1.72 V versus RHE and 1.1 to 1.8 V versus RHE, respectively. Every 20 hXAS spectra collected in 10 s during CV measurement were averaged into one spectrum during the data analysis. After the CV measurement, the same samples were used for CP measurement, which was performed for 60 s for each current step at different constant current densities. Every 40 hXAS spectra collected in 20 s during CP measurement were averaged into one spectrum during the data analysis.

### The dissolution of Co in different electrolytes

To monitor the dissolution of Co, we applied the same protocol used for the operando hXAS characterization in a RDE set-up with 20 ml of electrolyte. New electrolytes were used for the CV and CP measurements, and the Co concentration in the different electrolytes was quantified by inductively coupled plasma optical emission spectroscopy (ICP-OES), which is outlined in Supplementary Table 5.

### The absorption edge and its shift

The absorption edge *E*_edge_ is determined using the following equation^28,51^:

1 $${{{E}}}_{{\rm{edge}}}=\frac{1}{{{\rm{\mu }}}_{2}-{{\rm{\mu }}}_{1}}{\int }_{\!\!{{\rm{\mu }}}_{1}}^{{{\rm{\mu }}}_{2}}{{E}}({\rm{\mu }}){\rm{d}}{\rm{\mu }}$$

where *E*(*μ*) is the energy at the specific normalized absorption intensity and *μ*_1_ = 0.2 and *μ*_2_ = 1 are the lower and upper limits of the normalized intensity. Then the shift at the edge is as follows:

2 $${\Delta {{E}}}_{{\rm{edge}}}={{{E}}}_{{\rm{edge}}2}-{{{E}}}_{{\rm{edge}}1}$$

where *E*_edge1_ and *E*_edge2_ are the *E*_edge_ at the initial state at *t*_1_(*V*_1_, *i*_1_) and another state at the operating time *t*_2_(*V*_2_, *i*_2_), respectively.

## Online content

Any methods, additional references, Nature Portfolio reporting summaries, source data, extended data, supplementary information, acknowledgements, peer review information; details of author contributions and competing interests; and statements of data and code availability are available at 10.1038/s41557-025-01784-1.

## Supplementary information

Supplementary Information Supplementary Figs. 1–33, Notes I–VI and Tables 1–5.

## Source data

Source Data Fig. 1 Source data for Fig. 1.

Source Data Fig. 2 Source data for Fig. 2.

Source Data Fig. 3 Source data for Fig. 3.

Source Data Fig. 4 Source data for Fig. 4.

Source Data Fig. 5 Source data for Fig. 5.

## Data Availability

The data supporting the findings of this study are available within the Article and its Supplementary Information files. They have also been deposited in the Materials Cloud Archive at 10.24435/materialscloud:33-ch. The raw data files in another format are available from the corresponding authors upon reasonable request. Source data are provided with this paper.
