# Supplementary material for: Oxidation of interfacial cobalt controls the pH dependence of the oxygen evolution reaction
Source: Nat Chem. 2025 Mar 28;17(6):856–64. doi: 10.1038/s41557-025-01784-1 (PMC12141032; doi:10.1038/s41557-025-01784-1)
Supplement: Supplementary file 1 — Supplementary Figs. 1–33, Notes I–VI and Tables 1–5. [file 41557_2025_1784_MOESM1_ESM.pdf]

nature chemistry

Supplementary information https://doi.org/10.1038/s41557-025-01784-1

Oxidation of interfacial cobalt controls the pH dependence of the oxygen evolution reaction

In the format provided by the authors and unedited

Contents

Materials and catalyst 3
Supplementary figures and Tables 4
Supplementary Figure 1. 4
Supplementary Figure 2. 5
Supplementary Figure 3. 6
Supplementary Table 1. 7
Supplementary Figure 4. 8
Supplementary Table 2. 9
Supplementary Figure 5. 10
Supplementary Figure 6. 11
Supplementary Table 3. 12
Supplementary Figure 7. 13
Supplementary Figure 8. 14
Supplementary Figure 9. 15
Supplementary Note I. Frequency dispersion of interfacial capacitance. 16
Supplementary Figure 10. 17
Supplementary Figure 12. 19
Supplementary Figure 13. 20
Supplementary Figure 14. 21
Supplementary Note II. Protocol for the operando hXAS characterizations. 22
Supplementary Figure 15. 22
Supplementary Figure 16. 23
Supplementary Figure 17. 24
Supplementary Figure 18. 25
Supplementary Figure 19. 26
Supplementary Figure 20. 27
Supplementary Figure 21. 28
Supplementary Table 4. 29
Supplementary Figure 22. 30
Supplementary Figure 23. 31

Supplementary Figure 24. 32
Supplementary Note III. Discussion of the various processes contributing/influencing the final Co average oxidation state 33
Supplementary Figure 25. 33
Supplementary Figure 26. 34
Supplementary Figure 27. 36
Supplementary Figure 28. 37
Supplementary Figure 29. 38
Supplementary Note IV. Co dissolution in acidic environments. 39
Supplementary Table 5. 39
Supplementary Figure 30. 41
Supplementary Figure 31. 42
Supplementary Note V. Influence of rotation on the local pH and OER performance in phosphate buffer (pH = 7). 43
Supplementary Figure 32. 43
Supplementary Note VI. Discussions on the hXAS data analysis 45
Supplementary Figure 33. 45
References in Supplementary Information 46

Materials and catalyst.

The nano CoO_x (99.99%) used herein is commercially available from Sigma Aldrich. The KOH (99.9%), Nafion (99.9%), KH_2PO_4 (98%), K_2HPO_4 (98%), H_2SO_4 (95-97%), 2-Propanol (99.5%) were also bought from Sigma Aldrich, Germany.

Supplementary figures and Tables

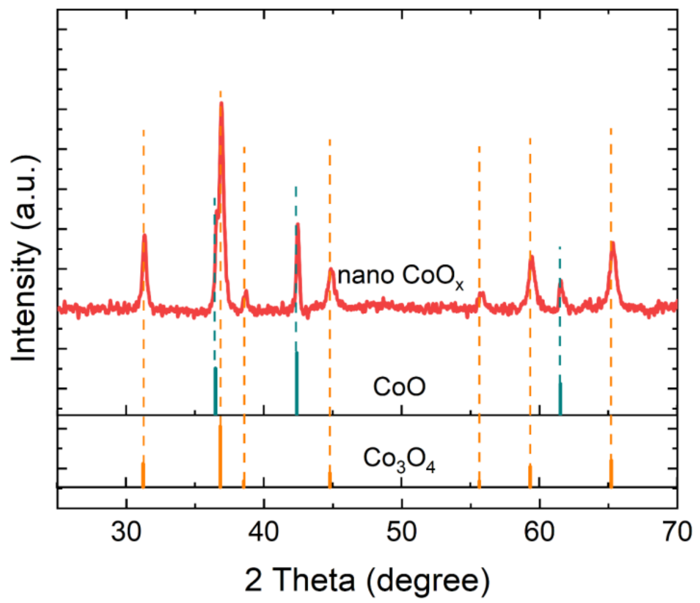

Supplementary Figure 1. (a) The XRD patterns of the commercial nano CoO_x, with the standard patterns of Co_3O_4 spinel structure and CoO rock salt structure as the references.

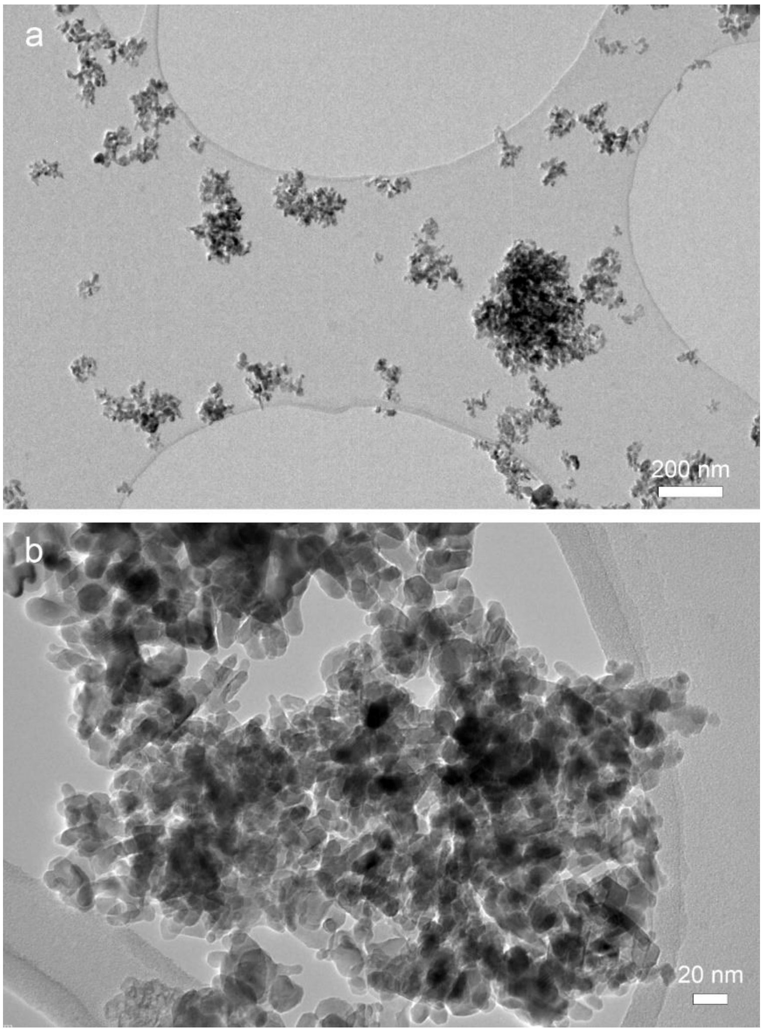

Supplementary Figure 2. TEM images of the nano CoO_x sample with the scale bars of (a) 200 nm and (b) 20 nm, respectively.

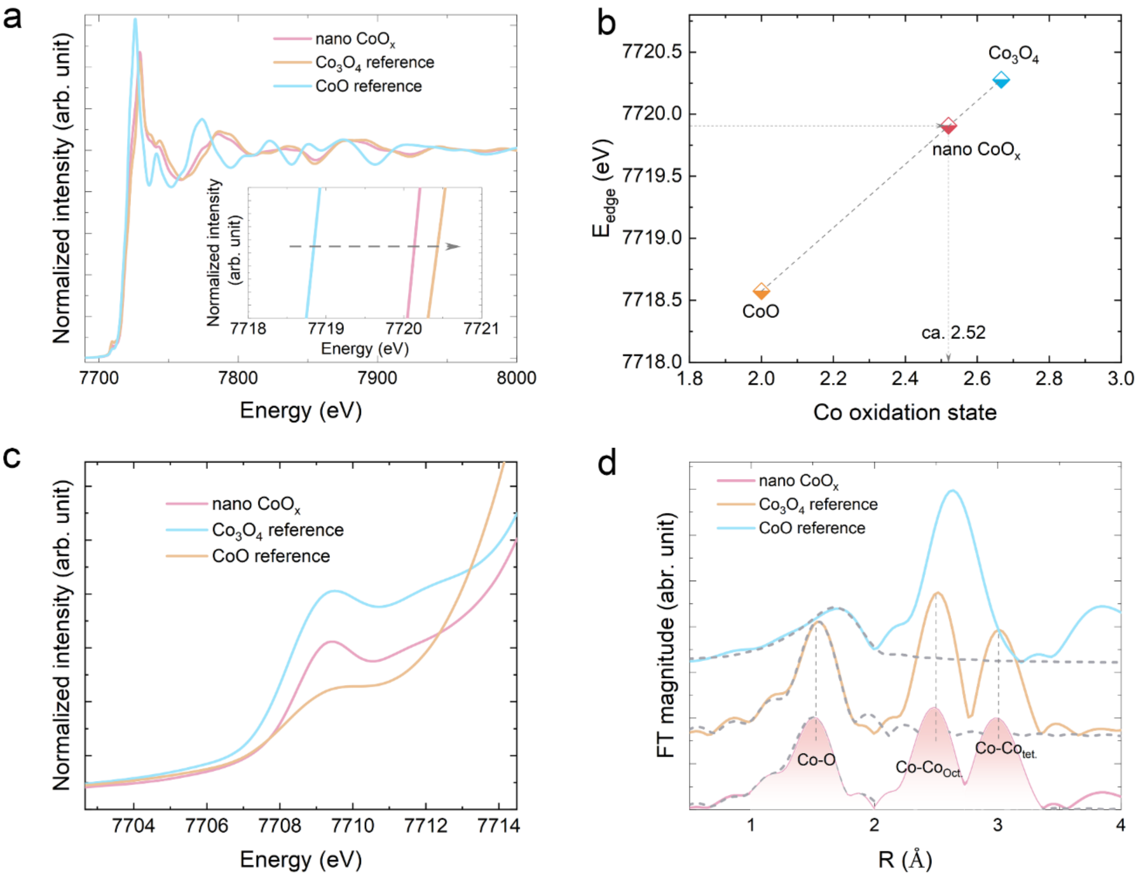

Supplementary Figure 3. Hard X-ray adsorption spectroscopy (hXAS) characterizations. (a) XANES spectra at the Co K-edge of nano CoO_x, Co_3O_4 and CoO reference. The inset highlights the shift in adsorption edges. (b) The adsorption energies of Co_3O_4 and CoO references are plotted as a function of Co oxidation state to estimate the average Co oxidation of nano CoO_x as ~2.52+. (c) The pre-edge features for the nano CoO_x, Co_3O_4 and CoO references. (d) The corresponding Fourier-transformed extended X-ray absorption fine structure (EXAFS) spectra of the Co K-edge. The fitting results for the first cell are shown as dashed lines.

Supplementary Table 1. The fitting parameters of the EXFAS spectra on the nano CoO_x, Co_3O_4 and CoO references. The CN, E_o, Reff, R and σ^2 are the coordination number, energy shift, theoretical Co-O bond distance, fitting Co-O bond distance and Debye Waller factor, respectively. During the fitting, the amplitude-reduction factor is set to be 0.82. A R-factor smaller than 0.02 suggests a good quality fit.

	CN	E_o (eV)	Reff (Å)	R (Å)	σ^2(Å^2)	R-factor
CoO reference	5.98 ± 0.51	2.73 ± 0.98	2.15	2.12 ± 0.01	0.0111 ± 0.0017	0.003
Co_3O_4 reference	3.78 ± 0.16	2.38 ± 0.45	1.92	1.92 ± 0.01	0.0092 ± 0.0038	0.002
Nano CoO_x	3.70 ± 0.24	-1.42 ± 0.85	1.92	1.91 ± 0.01	0.0031 ± 0.0007	0.003

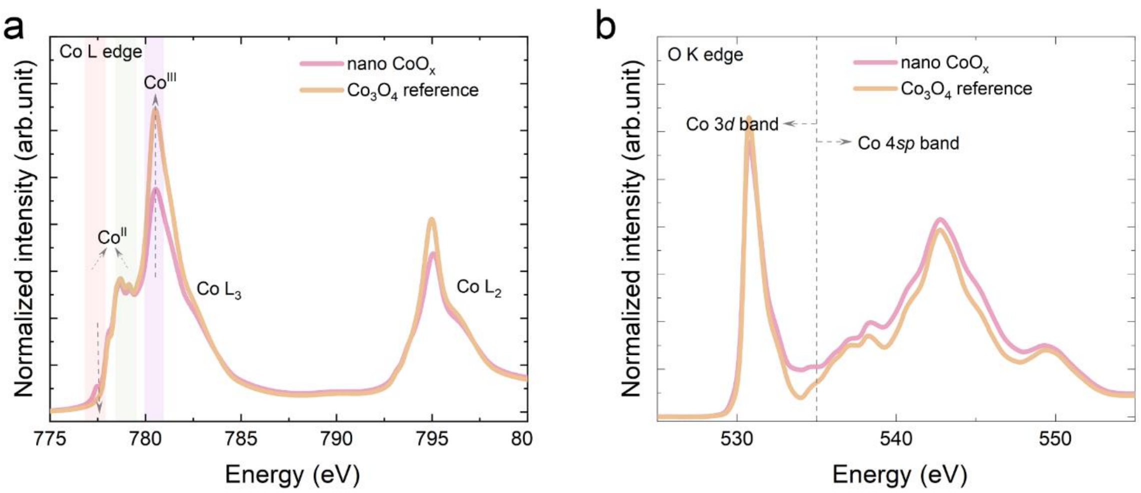

Supplementary Figure 4. Soft X-ray adsorption spectroscopy (sXAS) characterizations of the (a) Co L and (b) O K edges. The sXAS spectra were collected in the total electron yield mode[1] at the Phoenix beamline at the Paul Scherrer Institute. The peaks related to the Co^{II} (~780 eV) and Co^{III} species (~777 and 779 eV) in Co L_3 spectra are identified with different colored bands. The O K-edge spectra of nano-sized CoO_x is dominated by the low-spin Co^{III} features in the region related to the Co 3*d* band (<535 eV)

Supplementary Table 2. Summary of the peak intensities of Co L_2 and L_3 edges and the corresponding branching ratio $I(L_3)/[I(L_3) + I(L_2)]$.

	I (L3)	I (L2)	I(L3)/[I(L3) + I(L2)]
CoO	3.83	2.68	0.59
Nano CoOx	4.75	3.37	0.58
Co3O4	6.44	4.11	0.61

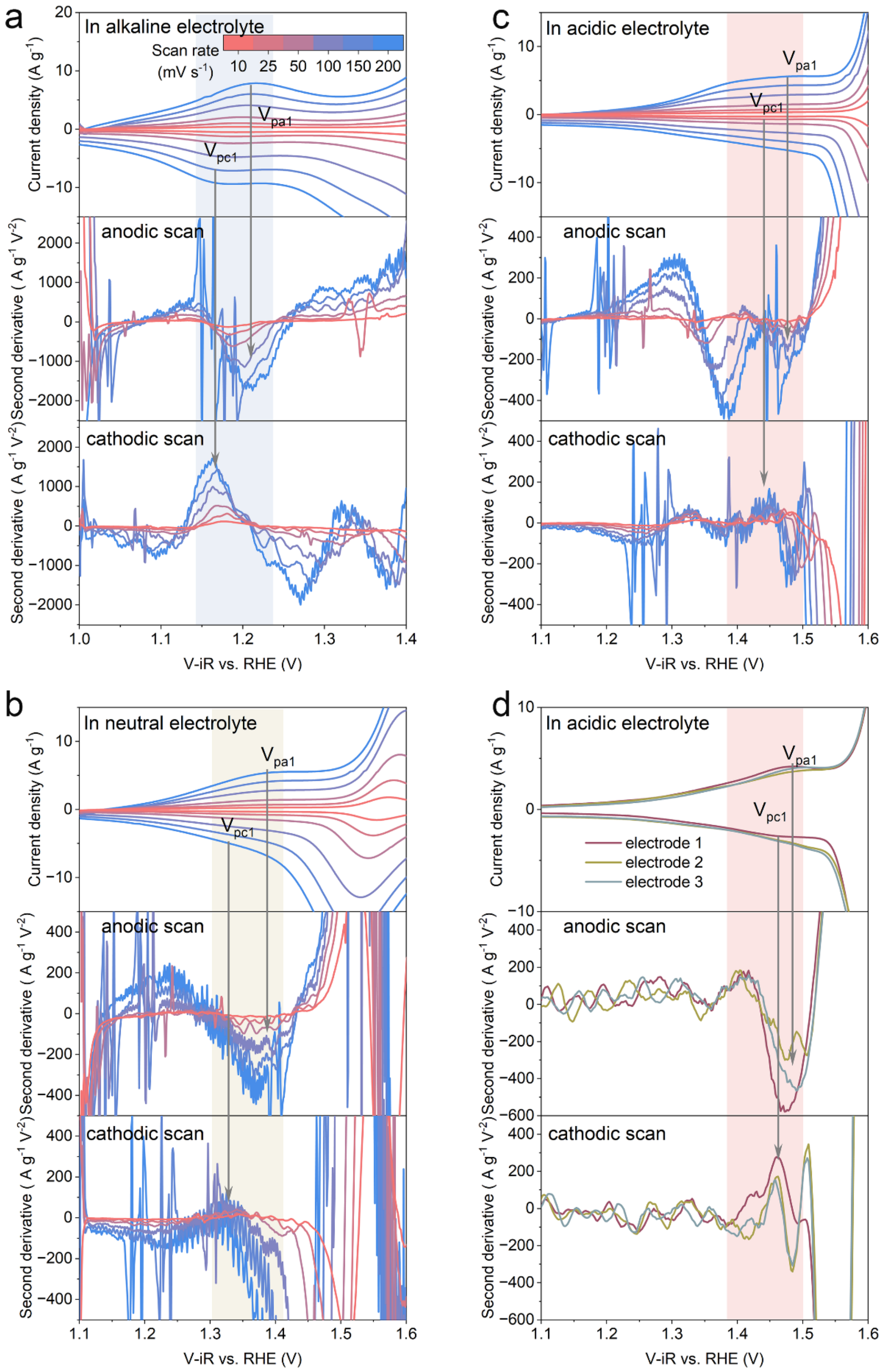

Supplementary Figure 5. Determining the peak positions for the $Co^{II/III}$ redox process by the second derivative of the CV curves in (a) alkaline, (b) neutral and (c) acidic electrolytes. V_{pa1} and V_{pc1} represent the peak positions at anodic and cathodic scan respectively. (d) As the peak features in the CVs are less significant in the acidic electrolyte, the CVs have been recorded with an additional three drop-cast electrodes to extract the peak position with greater reliability.

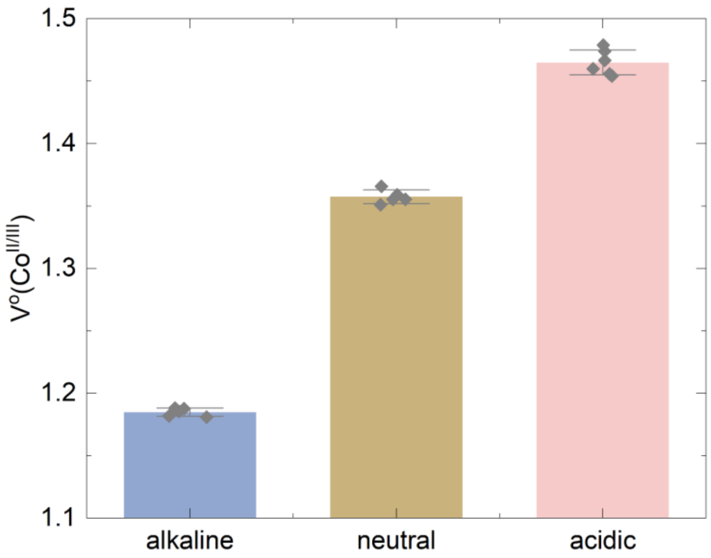

Supplementary Figure 6. The V°($Co^{II/III}$) determined by the second derivative of the CV curves. The standard deviation (SD) is obtained from averaging the data points collected at different scan rates. Specifically, in alkaline and neutral environments, the error bar represents the SD from averaging the data points (n = 5) collected at scan rates of 25-200 mV s^{-1}. In acidic environments, the error bar represents the SD from averaging 6 data points: three data points are collected in one electrode at the scan rates of 100-200 mV s^{-1}, and another three replicates were recorded with an additional three drop-cast electrodes at a scan rate of 100 mV s^{-1}, as shown Supplementary Figure 5. Data are presented as mean values +/- SD.

Supplementary Table 3. Summary of the $Co^{III/IV}$ redox peak position (V vs. RHE, iR-corrected) at different scan rates and in different electrolytes.

	Scan rate (mV s^{-1})	V_{pa2}	V_{pc2}	$V^{o}(Co^{III/IV})$
In alkaline electrolyte	200	1.526	1.438	1.482
	150	1.514	1.444	1.479
	100	1.505	1.450	1.477
	50	1.491	1.457	1.474
	20	1.478	1.464	1.471
	10	1.473	1.465	1.469
In neutral eletrolyte	200	1.621	1.514	1.568
	150	1.614	1.521	1.567
	100	1.603	1.529	1.566
	50	1.591	1.541	1.566
	20	1.580	1.549	1.565
	10	1.573	1.555	1.564
In acidic eletrolyte	200	1.573	1.555	1.564
	150	1.631	1.602	1.617
	100	1.629	1.602	1.616
	50	1.626	1.604	1.615
	20	1.622	1.606	1.614
	10	1.619	1.608	1.614

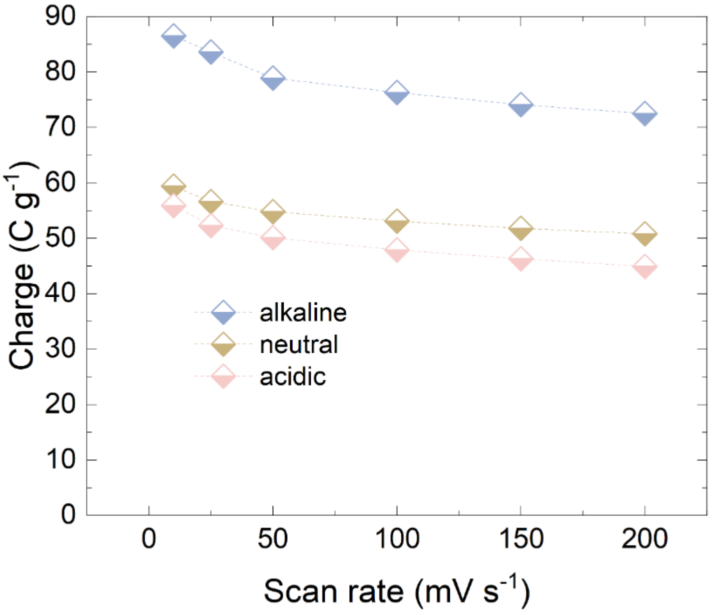

Supplementary Figure 7. The charge transferred during the two Co redox processes, which are calculated from the integrated area of the CV at different scan rate in Figure 2a. We note that the charge calculated herein is slightly over-estimated, with a small fraction charge contributed from the OER.

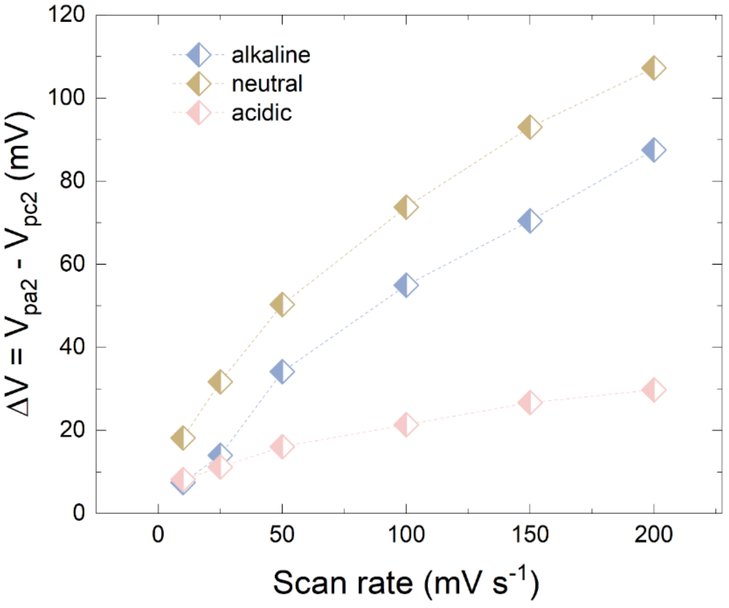

Supplementary Figure 8. The peak separation of V_{pa2} and V_{pc2} in the $Co^{III/IV}$ redox couple in different electrolytes.

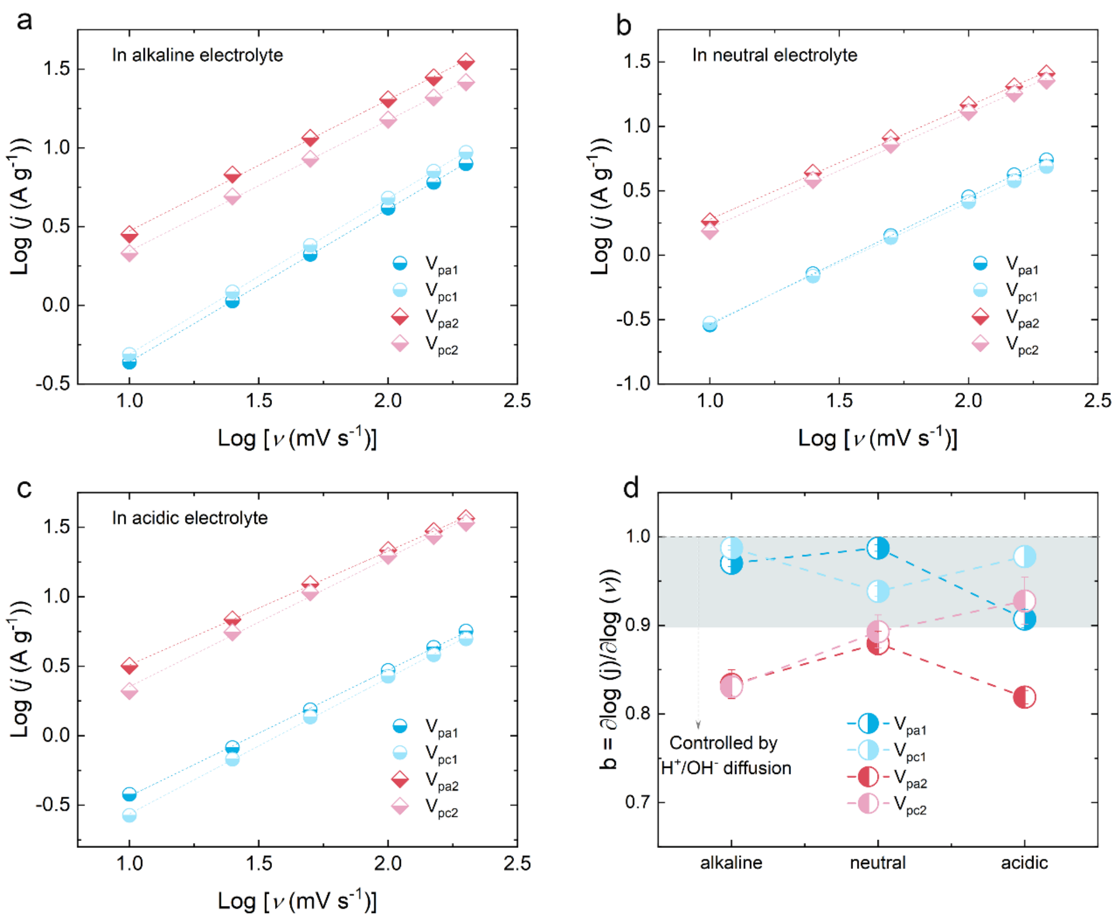

Supplementary Figure 9. Usually the redox peak intensity (j) and the scan rate (*v*) follow the power-law relationship ($j = av^b$).[2, 3] Theoretically, for *b* = 1 the redox process is a purely capacitive surface process, while *b* = 0.5 indicates that the process is diffusion-controlled[2, 3]. Plotting the logarithm of the Co redox peak intensity as a function of the scan rate extracts the *b* value in (a) alkaline, (b) neutral and (c) acidic electrolyte. The extracted *b* values are shown in (d), with the error bar from linear fitting.

Supplementary Note I. Frequency dispersion of interfacial capacitance.

The EIS spectra in Supplementary Figure 10 were collected using the Staircase Potentio Electrochemical Impedance Spectroscopy (Mott-Schottky) technique in the Biologic VMP-300 software (more details in Methods). In the literature, the M-S plot is usually extracted at the frequency of 1000 Hz. To show the influence of frequency on the capacitance and thus the V_{fb}, the M-S plots derived at 215, 464, 1000, 2154 and 4641 Hz have been studied.

There are two major capacitances at the (hydr)oxide/electrolyte interface, i.e., a double layer capacitance (C_{dl}) in series with a space charge capacitance (C_{sc}). Usually, the C_{dl} is one order of magnitude larger than the C_{sc}. Therefore, the serial capacitance C_s could be obtained by:

$$C_s^{-1} = C_{dl}^{-1} + C_{sc}^{-1} \approx C_{sc}^{-1} \quad (1)$$

In the EIS spectra, the electrical impedance (Z) is composed of two parts, namely the real part Re(Z) and the imaginary part Im(Z), and the latter one is related to the CPE and the interfacial capacitance[4, 5, 6]:

$$C_s = -1/(2\pi f * Im(Z)) \quad (2)$$

The C_s shows strong frequency dependence, which is commonly observed[7]. The frequency dispersion of C_s can be described as:

$$C_s = af^{n+1} \quad (3)$$

where *a* is constant, and *n* is the parameter related to frequency dispersion, which could be extracted and calculated from the slope in Supplementary Figure 12:

$$n = - 1/2*slope - 1 \quad (3)$$

Clearly, the frequency dispersion parameter *n* changes with applied potential (Supplementary Figure 12c). It is around -1.25 before the V_{fb} when there is a hole depletion layer at the interface. Then, it is shifted to around -2 after the V_{fb} when there is a hole accumulation layer at the interface. In addition, the frequency dispersion could be also affected by frequency dependence of the material dielectric constant or by the interfacial surface area[7]. Herein, the analysis of the frequency dispersion parameter suggests that the catalyst surface reconstruction is clearly associated with the $Co^{II/III}$ redox process. It has also to be noted that the reconstructed surface could have a different dielectric constant [8, 9]. Therefore, both changes of dielectric constant and surface area could synergistically contribute to the observed change of *n*.

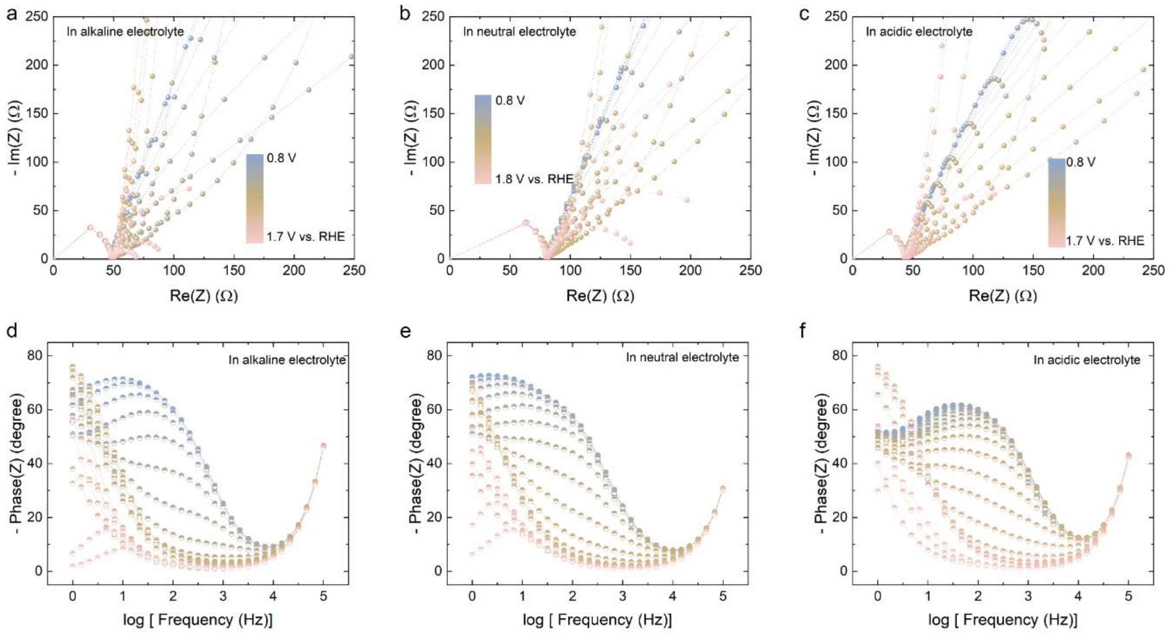

Supplementary Figure 10. Electrochemical impedance spectroscopy (EIS) at different potentials is used to extract the Mott-Schottky plot in Figure 3a and Supplementary Figure 11. Nyquist plots are shown for (a) alkaline, (b) neutral and (c) acidic electrolytes. (d-f) are the corresponding Bode plots.

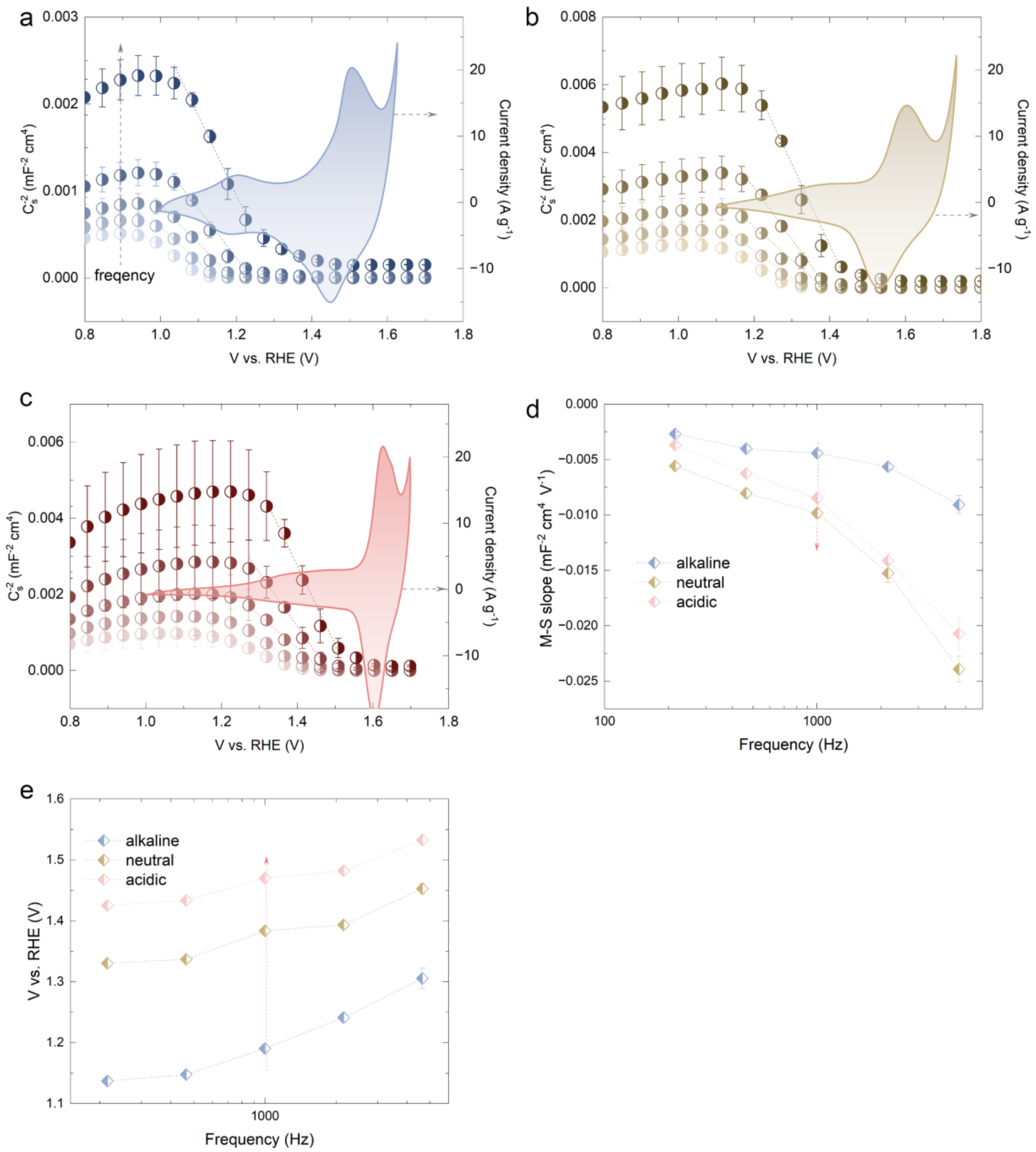

Supplementary Figure 11. (a-c) M-S plots derived at different frequencies in (a) alkaline, (b) neutral and (c) acidic electrolytes. The corresponding CV curves are shown for comparison. The error bar in (a-c) represents the SD obtained by averaging three replicates. Data are presented as mean values +/- SD (d) The flat band potentials (V_{fb}) and (e) M-S slopes that are extracted from the M-S plots shown in (a-c). Both V_{fb} and M-S slopes are slightly varied due to the frequency-dispersion of C_s. Error bars in (d-e) are obtained from linear fitting.

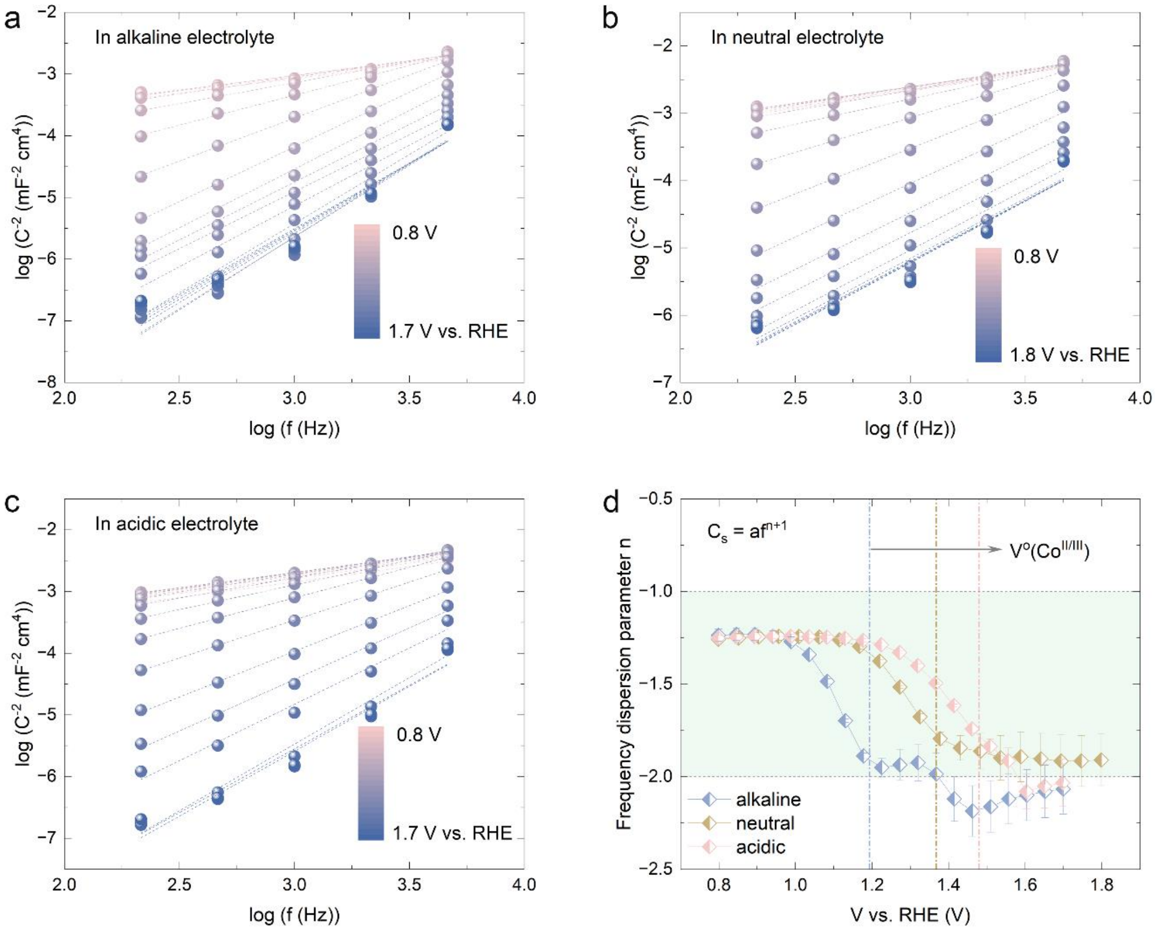

Supplementary Figure 12. Frequency dispersion of C_S. The log(C_S^{-2}) at different potentials is plotted as a function of log(*f*) to extract the slope = -2(*n*+1) in (a) alkaline, (b) neutral and (c) acidic electrolytes. In particular, *n* is the frequency dispersion parameter, which is plotted as a function of the applied potential in (d). Error bars in (d) are obtained from linear fitting.

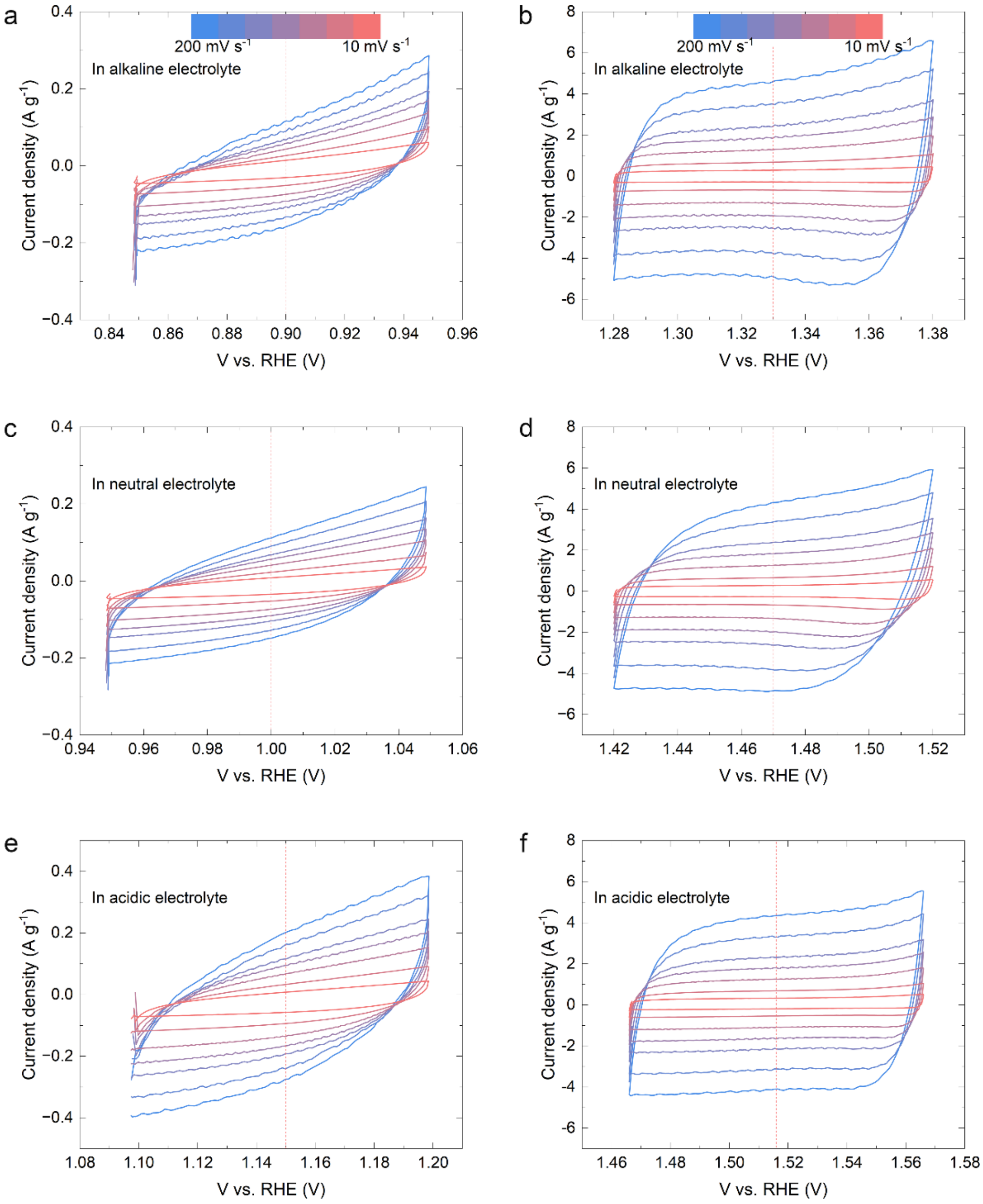

Supplementary Figure 13. CV curves collected before (a,c,e) and after (b,d,f) the V_{fb} process in (a-b) alkaline, (c-d) neutral and (e-f) acidic electrolytes. The scan rates are increased from 10, to 25, 50, 75, 100, 150 to 200 mV s^{-1}. Due to the shift of the $Co^{II/III}$ redox process in different electrolytes, the selected potential windows have been optimized accordingly.

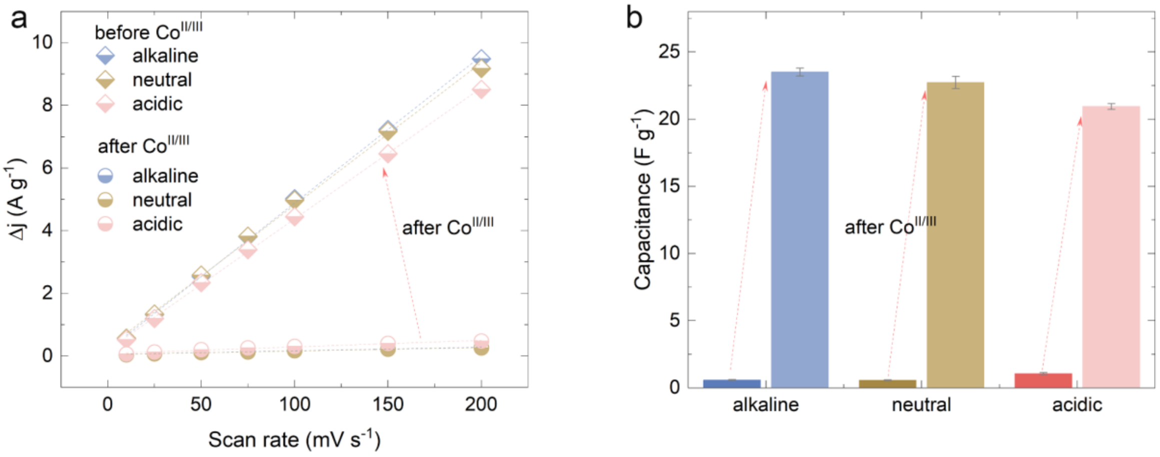

Supplementary Figure 14. (a) The difference in current density (Δj) between the anodic scan and cathodic scan was extracted from Supplementary Figure 13 and plotted as a function of the scan rates. The capacitance is half of the fitted slope. (b) Comparison of the capacitances extracted at the interface with hole depletion layer to those with hole accumulation layer in different electrolytes. Error bars are obtained from linear fitting.

Supplementary Note II. Protocol for the operando hXAS characterizations.

The experimental details for the hard X-ray adsorption spectroscopy (hXAS) characterizations can be found in the Methods. In this Supplementary Note, we demonstrate the protocol in Supplementary Figure 15 and make the connection between each step (step I to step IV) to the corresponding operando hXAS results.

In each electrolyte, we have used the same working electrode with CoO_x to run the electrochemical protocol (OCP → CV → OCP → CP) and simultaneously collected the operando hXAS spectra. A new working electrode is used for each electrolyte. The operando hXAS results collected during the CV measurement are shown in Figure 4. The comparison of operando hXAS results between the OCP before and after CV measurement is shown in Supplementary Figure 20. The total measuring time for two OCP measurements and 3 CV cycles is about 30 mins. Then the same electrode was used to do the CP measurement, and the corresponding hXAS results are summarized in Figure 5.

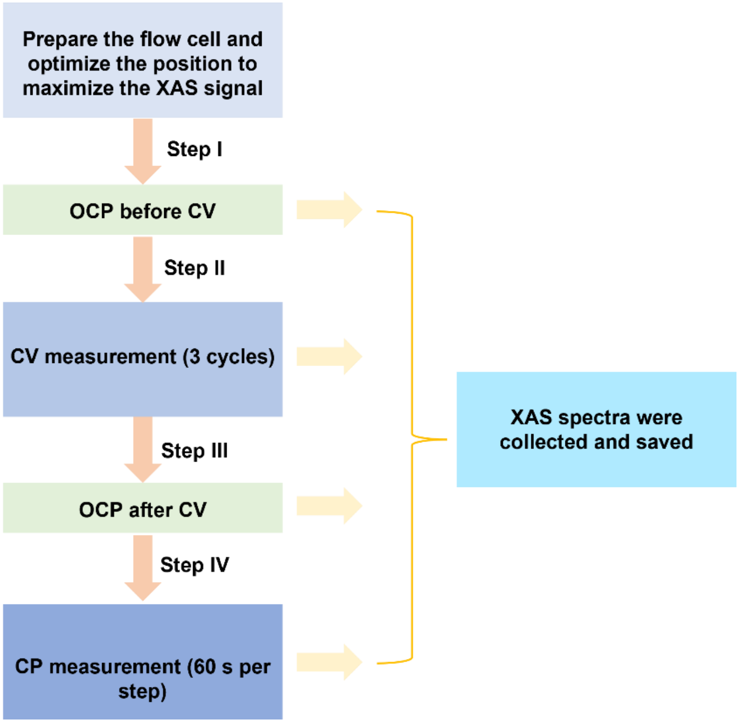

Supplementary Figure 15. Schematic demonstration of the protocol used for the operando hXAS characterizations.

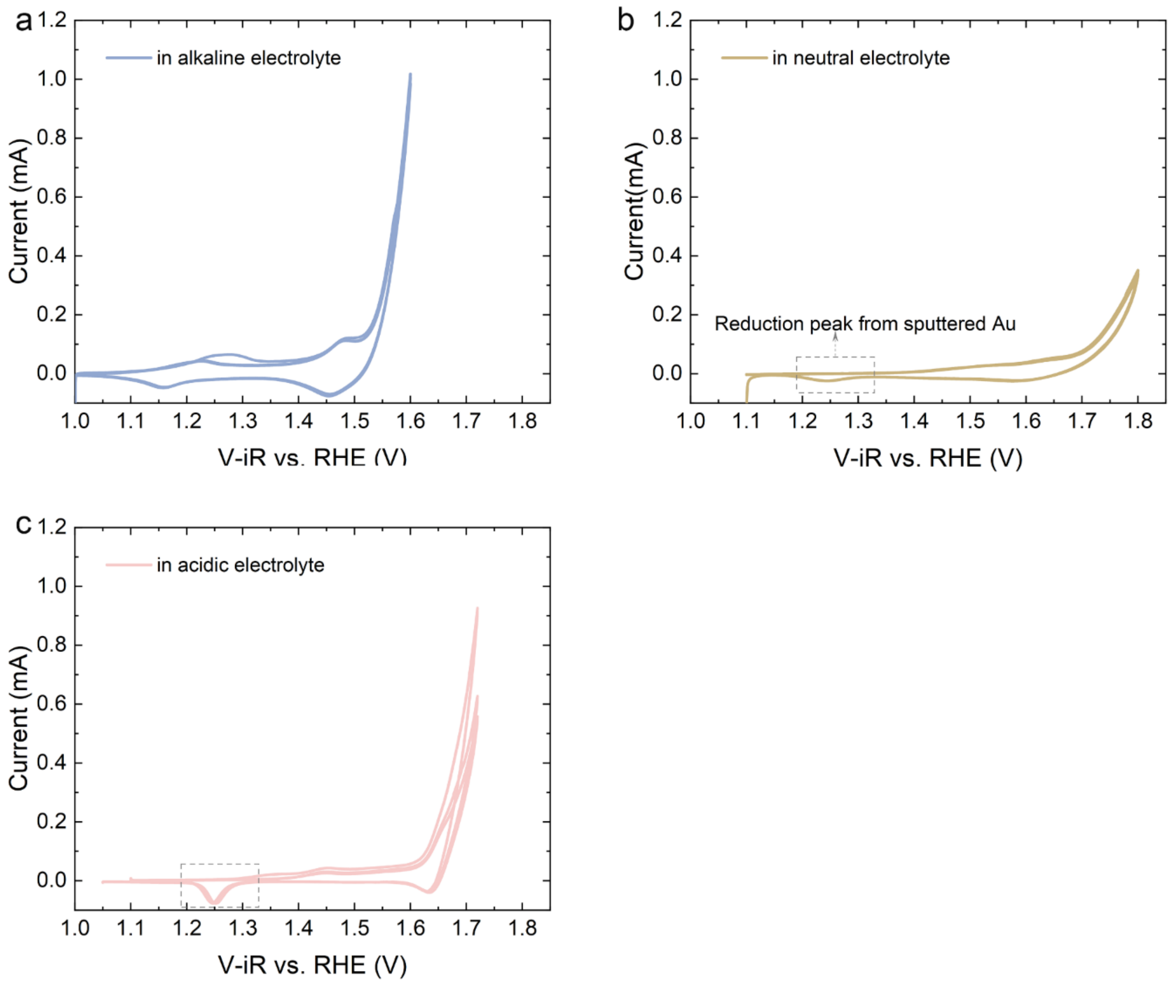

Supplementary Figure 16. The 3-cycle CV curves collected during operando hXAS characterizations in the flow cell in (a) alkaline, (b) neutral and (c) acidic electrolytes.

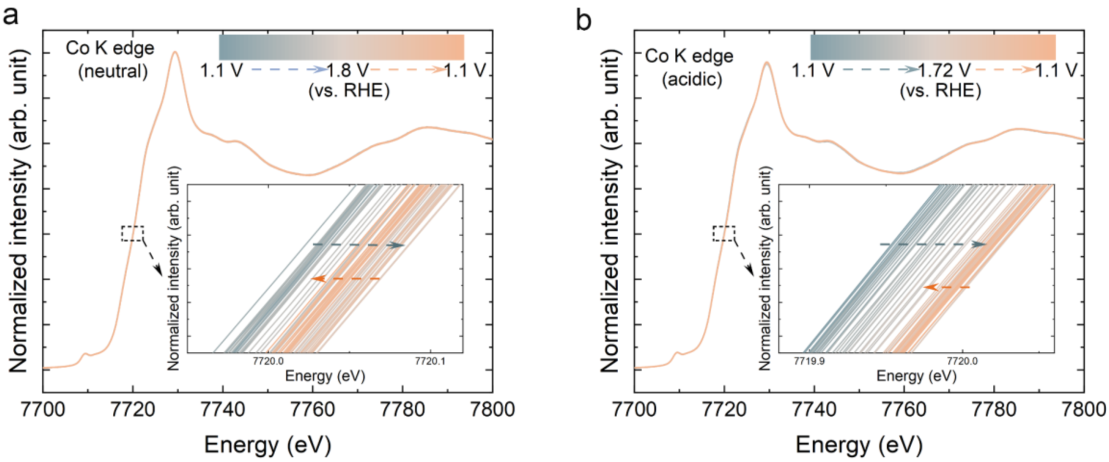

Supplementary Figure 17. The operando XANES spectra during CV measurement at the Co K-edge collected in (a) neutral and (b) acidic electrolyte, respectively.

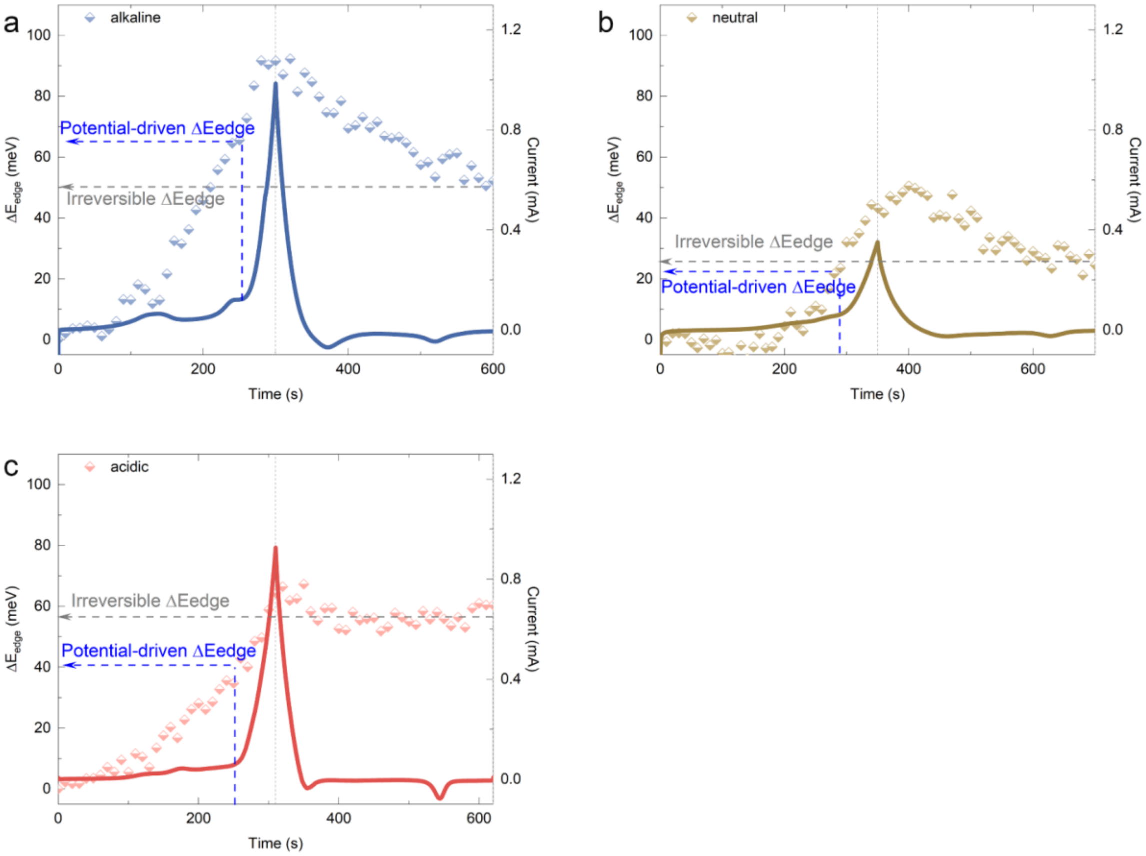

Supplementary Figure 18. The ΔE_{edge} observed during the 1st CV cycle in (a) alkaline, (b) neutral and (c) acidic environments, respectively. The data for full 3 CV cycles is outlined in Figure 4d-f.

The surface reconstruction can happen chemically when contact with electrolyte[10], and electrochemically driven by the applied potential or OER current. If the reconstruction can happen before the OER, then the increase of ΔE_{edge} can be observed before the obvious OER current. We define that this ΔE_{edge} is the potential-driven ΔE_{edge}.

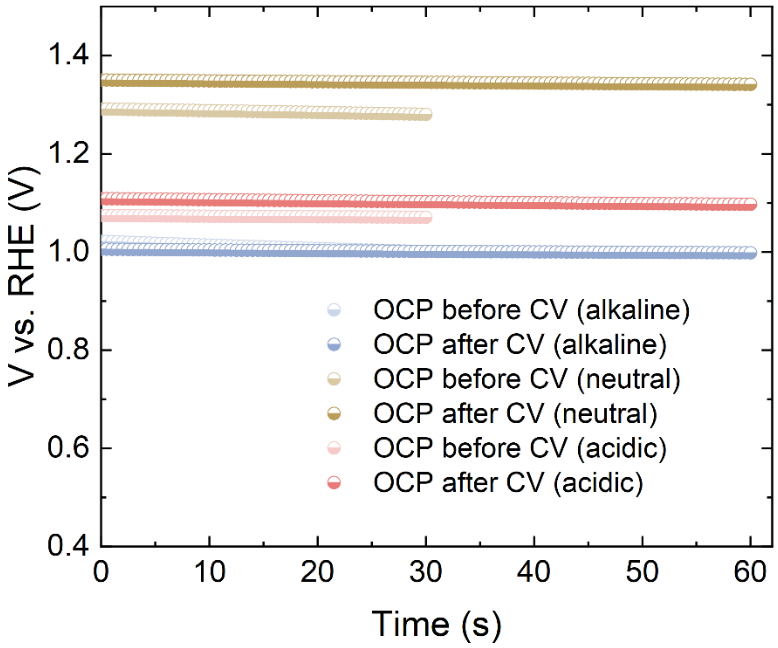

Supplementary Figure 19. The potential of the working electrode is plotted as a function of the time during open circuit potential (OCP) measurement, conducted before and after CV in the flow cell and in different electrolytes.

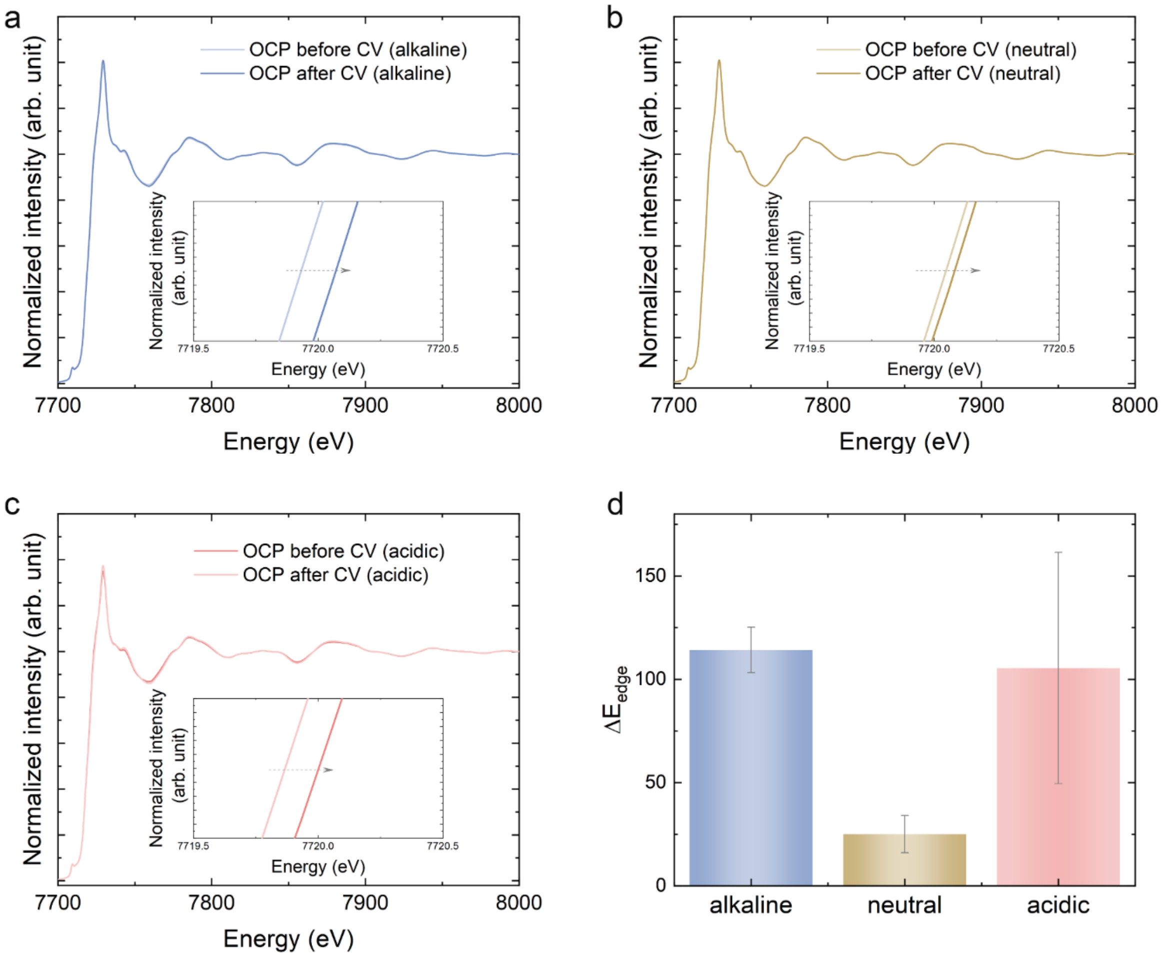

Supplementary Figure 20. Comparing the Co K-edge before and after CV measurements. The XANES spectra before and after CV measurement in (a) alkaline, (b) neutral and (c) acidic electrolyte. (d) The shift in adsorption energy (ΔE_{edge}) in different electrolytes. The error bars are from aligning the Co reference foil spectra for different measurements during the data analysis in the Athena software (see Supplementary Note VI for more details).

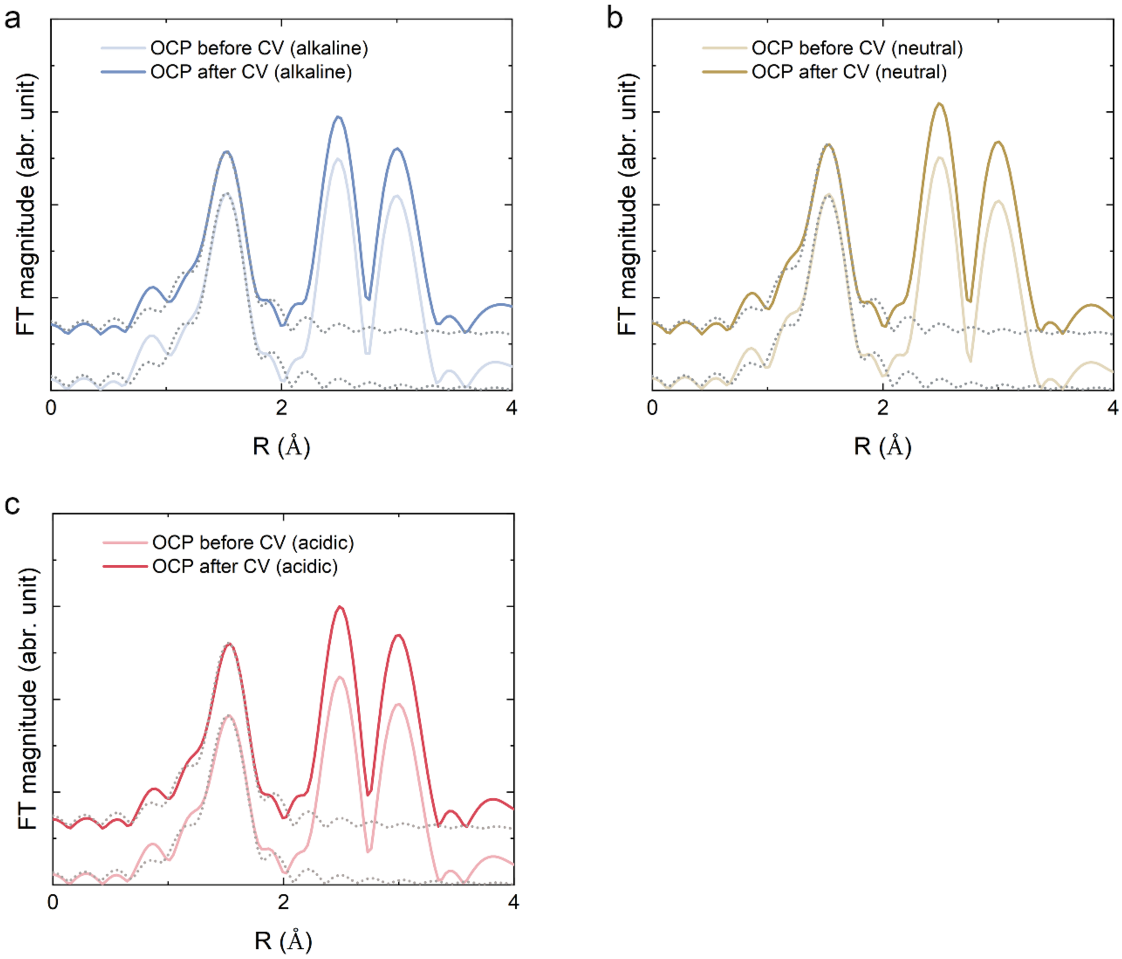

Supplementary Figure 21. Comparing the local bonding environment of the catalyst before and after CV measurements. The Fourier-transformed EXAFS spectra of the Co K-edge were collected in (a) alkaline, (b) neutral and (c) acidic electrolytes respectively. The dashed lines show the fitting results for the first shell to extract the information on Co-O bonding. The fitting results are summarized in Supplementary Table 4.

Supplementary Table 4. The fitting parameters of EXFAS spectra collected during the OCP measurement before and after 3 CV cycles. The CN, E_o, R and σ^2 are the coordination number, energy shift, fitting Co-O bond distance and Debye Waller factor, respectively. During the fitting, the amplitude-reduction factor is set to be 0.82. A R-factor smaller than 0.02 suggests a good quality fit.

	CN	E_o (eV)	R (Å)	σ^2(Å^2)	R-factor
OCP before CV (alkaline)	3.47 ± 0.18	-0.50 ± 0.67	1.91 ± 0.01	0.0021 ± 0.0006	0.003
OCP after CV (alkaline)	3.27 ± 0.20	-0.70 ± 0.78	1.91 ± 0.01	0.0022 ± 0.0007	0.004
OCP before CV (neutral)	3.40 ± 0.23	-0.30 ± 0.85	1.91 ± 0.01	0.0021 ± 0.0008	0.005
OCP after CV (neutral)	3.46 ± 0.23	-0.31 ± 0.85	1.91 ± 0.01	0.0023 ± 0.0008	0.005
OCP before CV (acidic)	3.14 ± 0.23	-0.92 ± 0.94	1.91 ± 0.01	0.0024 ± 0.0008	0.006
OCP after CV (acidic)	3.29 ± 0.19	-0.18 ± 0.73	1.91 ± 0.01	0.0022 ± 0.0007	0.004

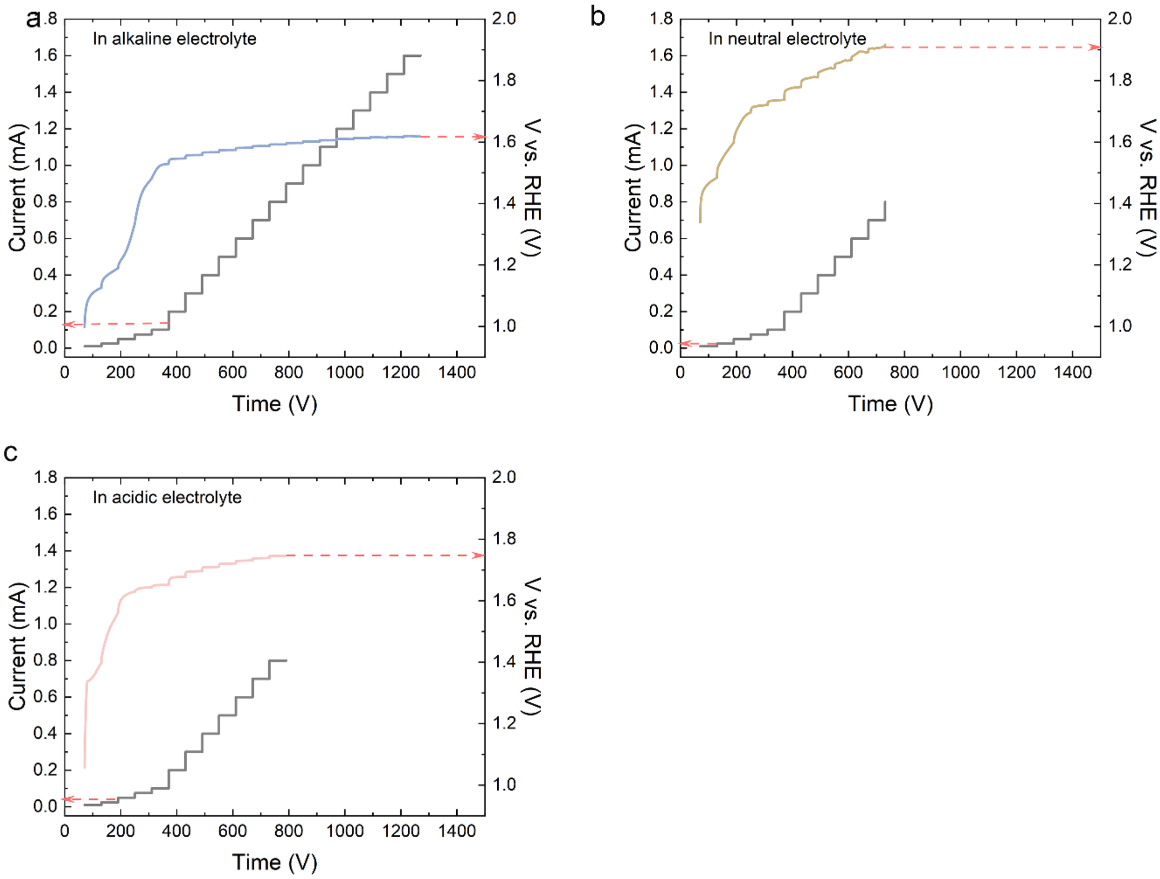

Supplementary Figure 22. The chronopotentiometry (CP) measurement conducted during the operando hXAS characterizations in the flow cell. The current and potential (vs. RHE) are plotted as functions of time, in (a) alkaline, (b) neutral and (c) acidic electrolytes respectively. The applied potentials are recorded every 0.5 s, to have the 120 data points for each step, which are used to generate the average and SD shown in Figure 5b.

The potential increases rapidly at low controlled currents ($i < 0.1$ mA) in all the three electrolytes, subsequently remaining quasi stable during each step with constant current ($i \geq 0.1$ mA).

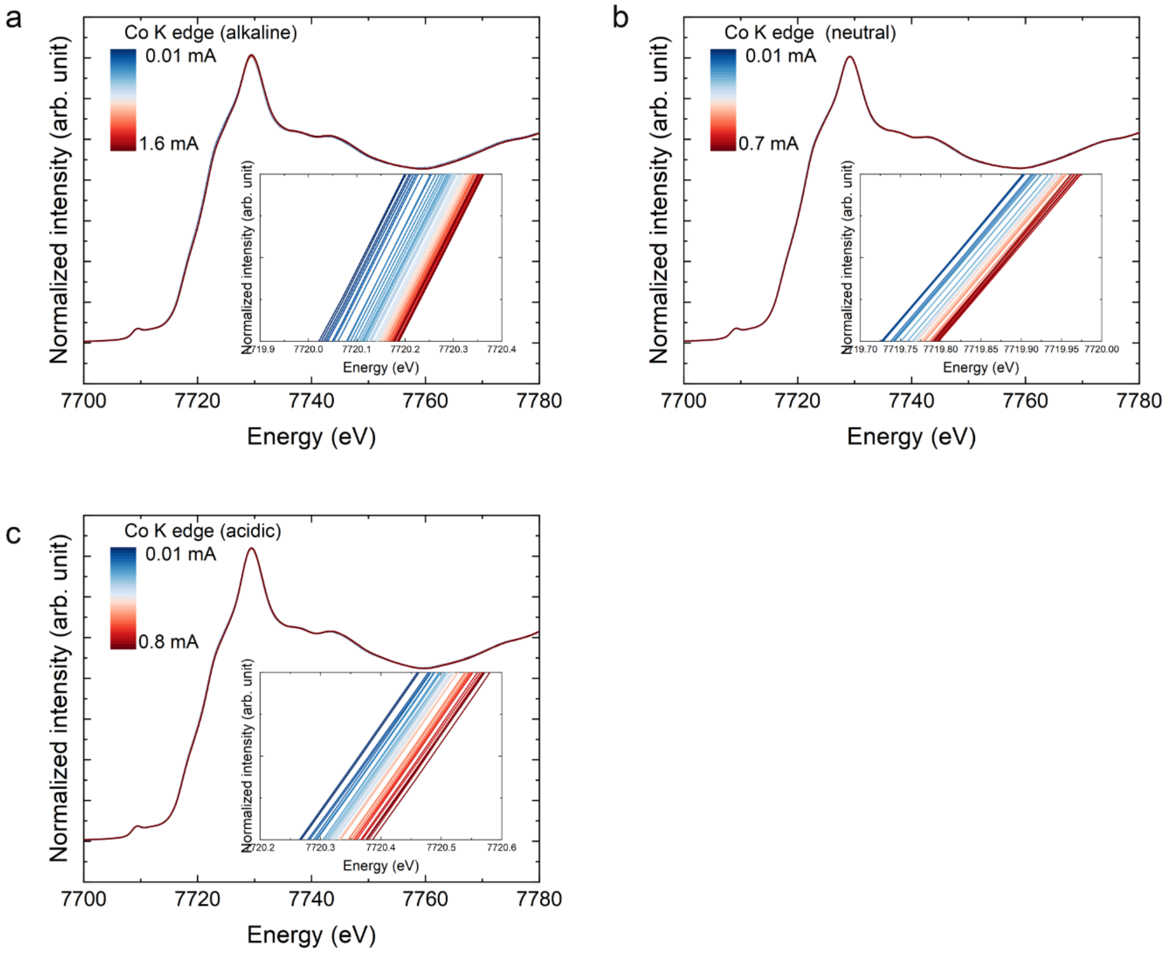

Supplementary Figure 23. The operando XANES spectra at the Co K-edge during the chronopotentiometry (CP) measurement in (a) alkaline, (b) neutral and (c) acidic electrolytes respectively.

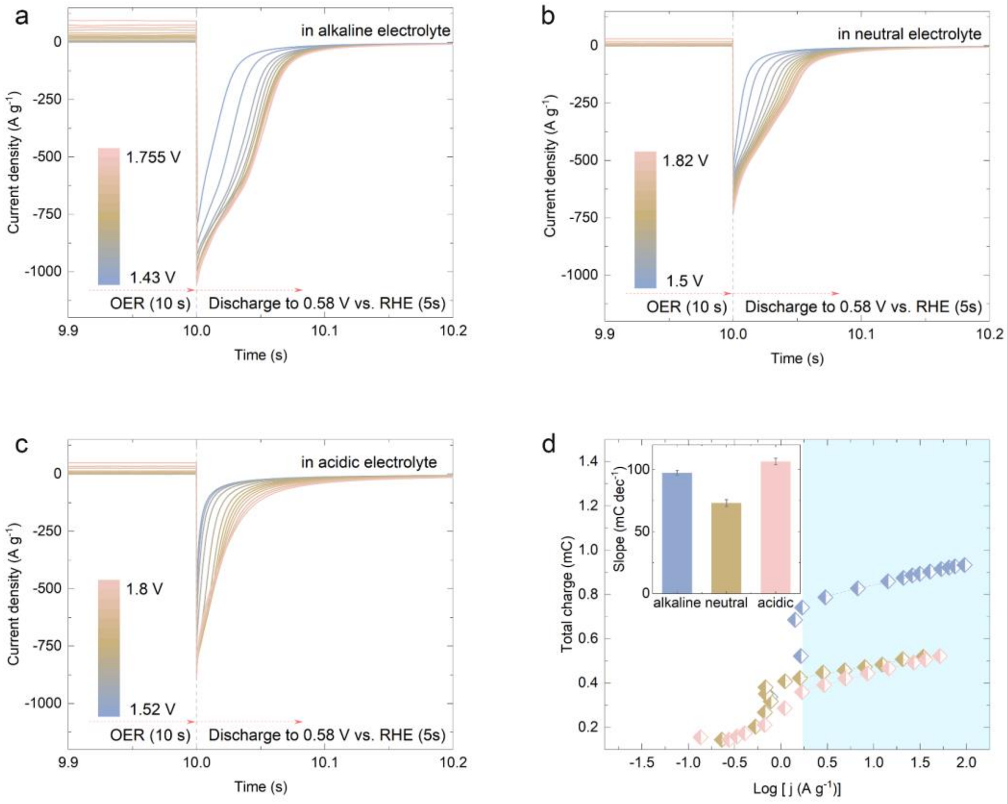

Supplementary Figure 24. Pulse voltammetry (PV) analysis in different electrolytes. The characterizations were performed on the rotating disk electrode setup. The working electrode with the catalyst is conditioned at different potentials for 10 s, and then was switched to 0.58 V vs. RHE for another 5s. The *j-t* curves were recorded in (a) alkaline, (b) neutral and (c) acidic electrolytes, respectively. (d) The current recorded during the last 2 s at high potential was averaged as the OER current, while the charge passed in the first 2 s after switching to 0.58 V vs. RHE was integrared as the total charge (i.e., including the surface charge and those from the electric double layer), to derive the plots; inset shows the slopes extracted from the linear region of the plots, with the error bars from linear fitting.

We adapted the pulse voltammetry (PV) analysis proposed by Nong et al.[11] to show the correlation between the charge vs OER current in Supplementary Figure 24. When the extracted total charge is plotted as a function of log(j(A g^{-1})), a linear correlation is observed in the region of high OER current (log(A g^{-1}) ≥ 0.25), as expected. The fitted slope is the smallest in a neutral electrolyte, indicating the change of total charge is also slower than in alkaline and acidic electrolytes. We note that before the linear region with low OER current (log(A g^{-1}) < 0.25), there is significant increase of the total charge, which should be related to the oxidation of the surface Co species.

Supplementary Note III. Discussion of the various processes contributing/influencing the final Co average oxidation state

The surface reconstruction processes for the CoO_x catalyst (mainly composed of Co_3O_4 structure) are schematically shown in Supplementary Figure 25, where the potential-driven surface reconstruction (Step 1) before the onset of OER and OER-coupled Co oxidation (Step2) are disentangled.

Step 1 represents the surface reconstruction from Co_3O_4 structure into a CoOOH-like structure[12], which happens at low applied potentials before the onset of the OER. The potential-driven ΔE_{edge} before the onset of the OER in Supplementary Figure 18 correlates with this process. In the CoO_x catalyst, irreversible changes associated to the observed Co edge shift (ΔE_{edge}) are consistently observed after 3 CV cycles in all the electrolytes, due to the formation of a thicker (oxy)hydroxide layer (Figure 4). Furthermore, the change of surface species in Co-based oxides can happen upon contact with water or electrolyte[13]. For the CoO_x catalyst here, an increase of the Co^{III} speces is also observed after ink preparation (discussed further in Supplementary Figure 30). In general, oxidized Co^{III} species are formed and stabilized at the catalyst/electrolyte interface when the catalyst is exposed to the water or electrolyte. It is not a fully reversible transformation to the initial state when the potential is scanned back (e.g., back to 1.1 V vs. RHE), resulting in the observed irreversible ΔE_{edge} after CV measurements.

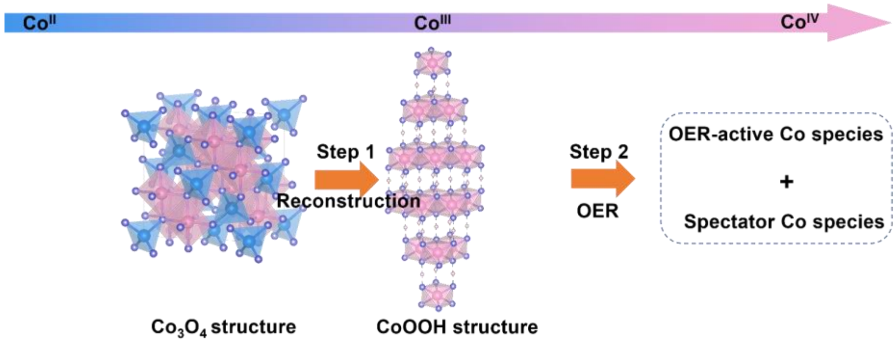

Supplementary Figure 25. Sketch illustrating the reconstruction and oxidation process for Co_3O_4 structure (i.e., main component in CoO_x catalyst) before the occurring of the OER. Step 1 represents the potential-driven surface reconstruction to form a CoOOH-like structure before the OER, which is potentially pH-dependent. Further Co oxidation at Step 2 to generate both spectator Co species and OER-active Co species is driven by the OER current, showing a good linear relationship between ΔE_{edge} and log(i).

However, the oxidized Co^{III} species can be reduced back (at least partially) to the initial state when the Co-based catalysts are taken out from the electrolyte and dried. For example, even though significant irreversible ΔE_{edge} is observed after 3 CV cycles in alkaline and neutral electrolytes, the ex-situ soft XAS characterizations on the Co L edge reveal no significant difference in the Co species on the post-mortem samples (discussed further in Supplementary Figure 31). A decrease of peak intensity representing the Co^{III} species is observed in the sample after being tested in an acidic electrolyte, because the Co^{III} species are the active centers, oxidized into Co^{IV} species to drive the OER in an acidic electrolyte[14], resulting in partial dissolution. The soft XAS characterization at the Co L edge also suggests that the

surface reconstruction is pH dependent, consistent with the results found in the operando hXAS characterizations (Figure 4-5 and Supplementary Figure 18).

In Supplementary Scheme 2, Step 2 represents the further Co oxidation at the interface, which is driven by the OER current to show a good linear relationship between ΔE_{edge} and log(i), as demonstrated in Figure 5a. If only the OER-active species are generated in this process, the slope extracted in Figure 5a should be the same in different electrolytes. The slopes change when varying the electrolyte, indicating the formation of OER-active Co species is accompanied by the oxidation of spectator Co species, which is also pH dependent. Therefore, we note that the surface reconstruction and the Co species oxidation at the interface is potential-driven before the OER onset, later it is coupled with OER. Both processes are influenced by the electrolyte pH.

Beside the pH-dependence, Haase et al. proved that Co oxidation state change is influenced by the nanoparticle size[15]. A smaller nanoparticle results in a larger change of average Co oxidation state under OER conditions. More importantly, it is also very clear that even after the activation, the change of average Co oxidation state from the activated stage to the stage under OER conditions is still size-dependent. In addition, not all the oxidized Co will drive the OER, a proportion of oxidized Co species act as spectators.

In this work, the particle size of CoO_x is around 20-40 nm, much larger than those used by Haase et al. (1~9 nm)[15], therefore it is difficult to fully activate them. In addition to the pH-dependent surface reconstruction and inferior stability of CoO_x in acidic electrolyte, it is difficult to pre-activate the CoO_x to reduce the influence of the spectator Co species. Note that, the catalysts with larger nanoparticle size (i.e., lower specific area) are less likely to be fully activated, and therefore will have lower contribution of the spectator Co species. Accordingly, we complemented the operando XAS results with a Co_3O_4 control sample in acidic electrolyte, which has a smaller BET (Brunauer–Emmett–Teller) specific surface area of ~21 $m^2\ g^{-1}$ compared to the ~31$m^2\ g^{-1}$ for the nano-size CoO_x. The Co_3O_4 control sample shows a similar CV profile as the CoO_x in acidic environment (Supplementary Figure 26). The redox peak intensity is lower in the Co_3O_4, due to lower specific surface area compared to CoO_x.

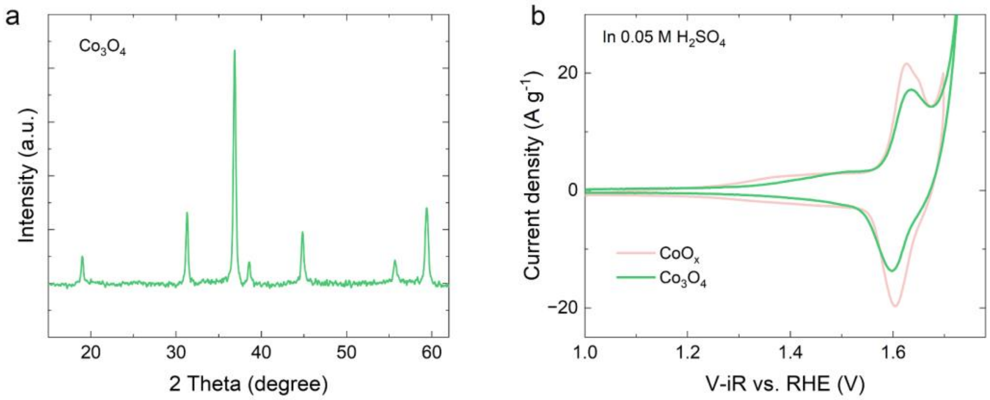

Supplementary Figure 26. The Co_3O_4 control sample was synthesized by a two-step protocol, including the preparation of $Co(OH)_2$ by a wet-chemistry precipitation[16] and then the conversion to Co_3O_4 by annealing the $Co(OH)_2$ at 450 °C for 2h in air. (a) The structure of Co_3O_4 is confirmed by XRD characterization. (b) It also shows similar redox peaks as the CoO_x

in 0.05 M H_2SO_4 electrolyte. The reduced redox peak intensity in Co_3O_4 results from lower specific surface area compared to the CoO_x (20 m^2 g^{-1} vs. 31 m^2 g^{-1}).

Additionally, a similar protocol for the operando hXAS characterization (outlined in Supplementary note II) has been applied to Co_3O_4 to reveal the Co oxidation state change in acidic electrolyte (Supplementary Figure 27a). An irreversible ΔE_{edge} of 15 meV is observed after 3 CV measurements (Supplementary Figure 27b), which is smaller than that found for CoO_x. Interestingly, this irreversible ΔE_{edge} is also comparable to the potential-driven ΔE_{edge} observed before the onset of OER, which is also consistent with the results found for the CoO_x in different electrolytes (Supplementary Figure 18), suggesting the irreversible oxidation of species at the interface after CV measurement correlates with the potential-driven surface reconstruction.

Furthermore, the ΔE_{edge} observed in the Co_3O_4 control sample, with a smaller specific surface area, during the steady state CP measurement is plotted as a function of log(i), to derive a linear relationship similar to that found for the CoO_x catalyst (Supplementary Figure 28). Note that the linear slope found in CoO_x is about 3 times larger than that in Co_3O_4 control sample, indicating that the accumulation of oxidized species (including both the spectator Co species and OER-active Co species) in CoO_x is faster with respect to the change in OER current It is easy to understand that the OER-active Co species at the interface should scale linearly with log(i) of the OER. The general linear relationship between total ΔE_{edge} and log(i) found for Co_3O_4 in acidic electrolytes and for CoO_x in different pH environments suggests that the generation of spectator Co species at the interface also scales linearly with the log(i). This reveals that the formation of spectator Co species after the onset of OER is coupled with the reaction.

Due to the similar chemical environment of the oxidized Co species, it is difficult to differentiate the different contributions to the overall Co oxidation change due to the bulk averaging characteristics of the hXAS technique. However, the formation of Co spectator coupled with OER and active species can be depicted by the linear slope of the ΔE_{edge} ~ log(i) plot, which show dependence on the pH environment of the electrolyte and the specific surface area of the catalysts. The different slopes extracted from the ΔE_{edge} ~ log(i) plots (in Supplementary Figure 28 and Figure 5a) enable the comparison of the different trends for the accumulation of oxidized Co species in different catalyst/electrolyte interfaces.

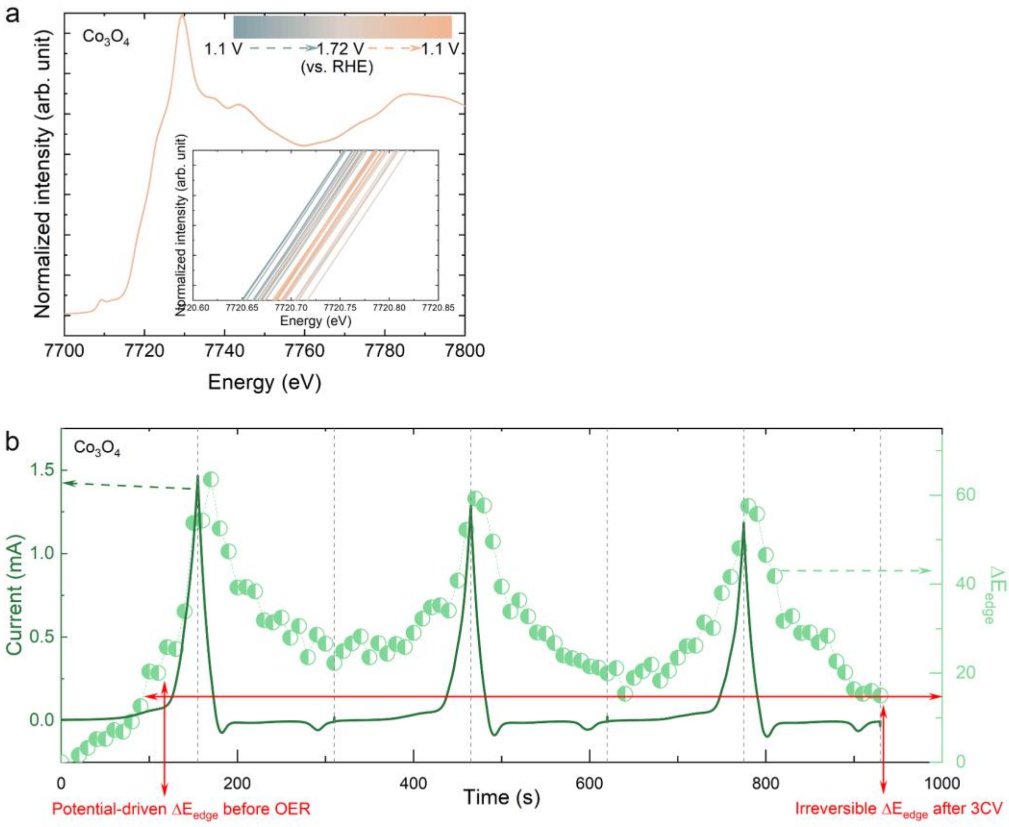

Supplementary Figure 27. (a) Operando hXAS characterization of Co_3O_4 at the Co K edge during CV measurement in an acidic electrolyte. Inset shows the edge shift in response to the applied potential. (b) The ΔE_{edge} (calculated by taking the E_{edge} at the start of the 1st CV as the initial state) observed during the CV measurement.

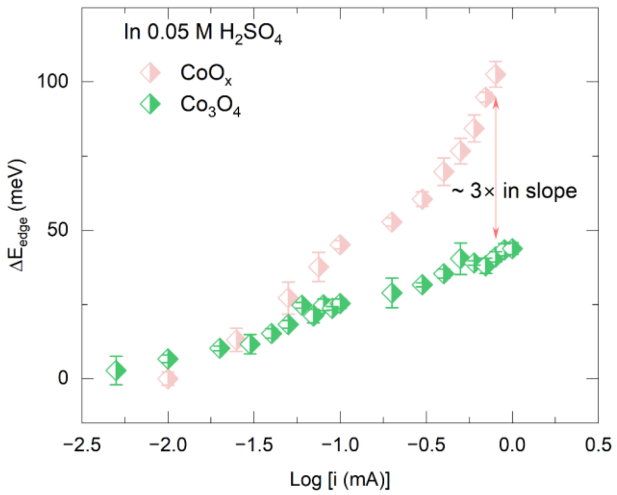

Supplementary Figure 28. Comparison of the ΔE_{edge} plotted against log(i) in the Co_3O_4 control sample with the CoO_x catalyst. The slope for CoO_x is around 3 times larger than that for Co_3O_4. Error bar represents the SD obtained by averaging the ΔE_{edge} of three spectra recorded for each step during CP measurement. Data are presented as mean values +/- SD.

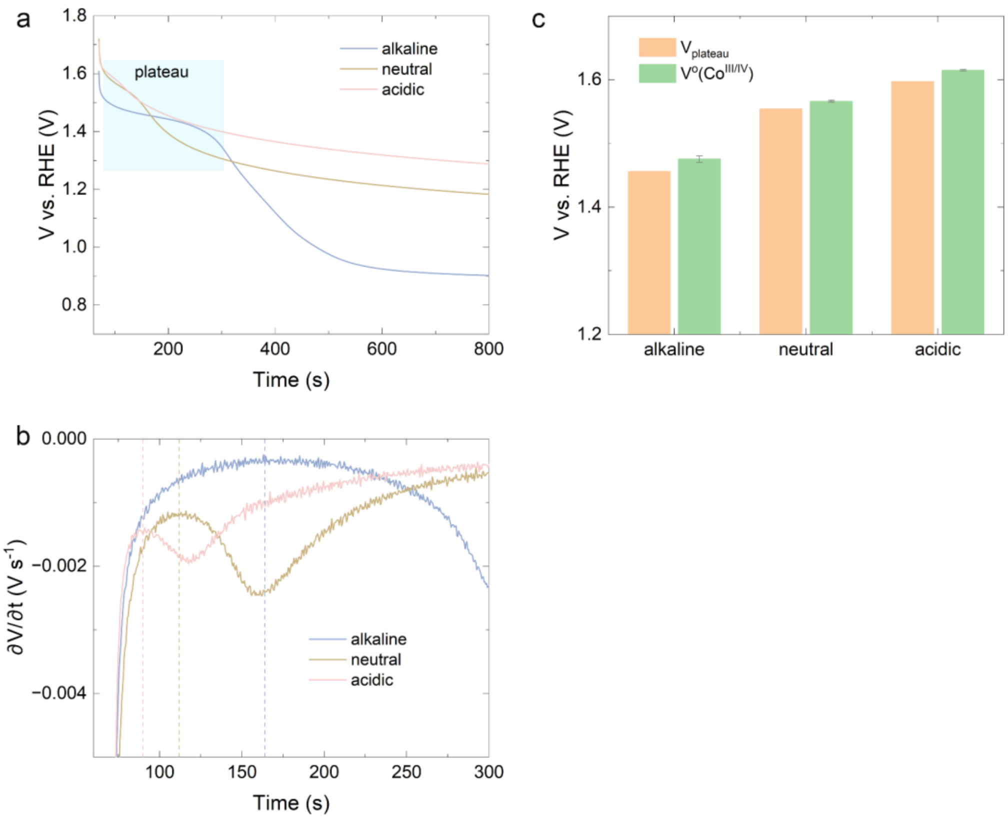

Supplementary Figure 29. Analysis of OCP collected at different electrolytes. (a) The *V-t* curves collected under the OCP conditions. The hold potential in each electrolyte before recording the OCP curves was higher than the corresponding $V^o(Co^{III/IV})$ extracted from CV measurements in Figure 2a. (b) The derivatives of the *V-t* curves to show the plateau features. (c) Comparison of $V_{plateau}$ and $V^o(Co^{III/IV})$ to show the correlation. The error bar for $V^o(Co^{III/IV})$ represents the SD obtained by averaging the 7 data points recorded at different scan rates, as detailed in Supplementary Table 3.

In Supplementary Figure 29, we have performed new measurements recording for about 15 min the open circuit potential of CoO_x in alkaline, neutral and acidic electrolytes after the catalyst was being held at an applied potential higher than the corresponding $V^o(Co^{III/IV})$ extracted from CV measurements in Figure 2a and with obvious OER current. We observe the plateau feature in every electrolyte, and it is more obvious in the derivative curve. The time for observing the plateau in each electrolyte can be determined from the derivative curve, as indicated by the vertical dashed curves, which decrease in a low pH environment. Correspondingly, we define the potential of the plateau ($V_{plateau}$) for each electrolyte. We find that the $V_{plateau}$ matches well with the corresponding $V^o(Co^{III/IV})$ in each electrolyte, highlighting the plateau should be related to the Co^{IV} to Co^{III} reduction. In addition, the observation of $V_{plateau}$ in the OCP measurement confirms that Co^{IV} species are formed in all the electrolytes. However, their longevity at the interface appears to be pH-dependent since it takes a shorter time to reach the observed plateau in a lower pH environment.

Supplementary Note IV. Co dissolution in acidic environments.

Supplementary Table 5. Summary of ICP-OES results on the electrolytes collected after 3 CV and CP measurements.

	Dissolved Co/ppm	Dissolved Co/mol	Stability number ($n(O_2)/n(Co)$)
Alkaline (3CV)	<	-	-
Alkaline (CP)	<	-	-
Neutral (3CV)	<>	-	-
Neutral (CP)	<>	-	-
Neutral (3CV, 0 rpm)	<	-	-
Neutral (CP, 0 rpm)	0.011	3.82	128
Acidic (3CV)	0.056	18.90	-
Acidic (CP)	0.024	8.27	59

Note: The total volume of electrolyte is 20 mL; the catalyst was first activated by running 3 CV cycles at the scan rate of 2 mV s^{-1}, and the electrolyte was collected. New electrolyte was used for the CP measurement. In neutral electrolyte, a control group performed without rotation of RDE (i.e., 0 rpm) has also investigated. < : below the detection limit of 0.003 ppm; <>: between the detection limit of 0.003 ppm and the quantification limit of 0.01 ppm.

In Supplementary Table 5, a significant Co dissolution is observed in the first 3 CV measurement before the CP measurement, partially due to the dissolution of loosely bound oxides at the beginning[17]. In addition, it is also attributed to the low stability of Co-based catalysts at a relatively lower applied potential. Firstly, Bloor et al. showed that the in-situ formed Co-oxide film will be dissolved back to the electrolyte under OCP conditions[18]. Secondly, Co_3O_4 catalysts suffer from a faster Co dissolution at OCP conditions than at anodically biased OER conditions at a constant current density of 10 mA cm^{-2} (ref.[12]). This results from the in-situ formation of CoOOH-like species, confirmed by in-situ Raman characterizations at around 1.2 V vs. RHE, close to the OCP. Thirdly, it is proposed that at the catalyst surface, a transition from the reconstructed CoOOH to CoO_2 at applied potentials above 1.8 V vs. RHE can enhance the catalyst stability[19]. Furthermore, we note that recently, Priamushko et al. have used online ICP-MS to reveal the transient dissolution processes in Co_3O_4 during OER in acidic environments[20]. It is pointed out that the OER-induced Co dissolution is actually lower than the initial contact dissolution. And the stability window for the formation of stable $Co^{III\text{-}IV}$ species is about 1.55 to 1.65 V vs. RHE.

Therefore, the Co-based catalysts could be less stable at applied potentials before OER, since the in-situ generated CoOOH-like species at the potential window of around 1.2 ~1.6 V vs. RHE are less stable than the pristine Co_3O_4 and the further oxidized Co^{IV} species in CoO_2. Herein, the potential window for the CV measurement

in acidic electrolyte is 1.1 to 1.72 V vs. RHE. Therefore, some of the in-situ reconstructed CoOOH-like species formed in the anodic scan could be dissolved when the applied potential is scanned back to 1.1 V vs. RHE, leading to significant Co dissolution during CV measurement.

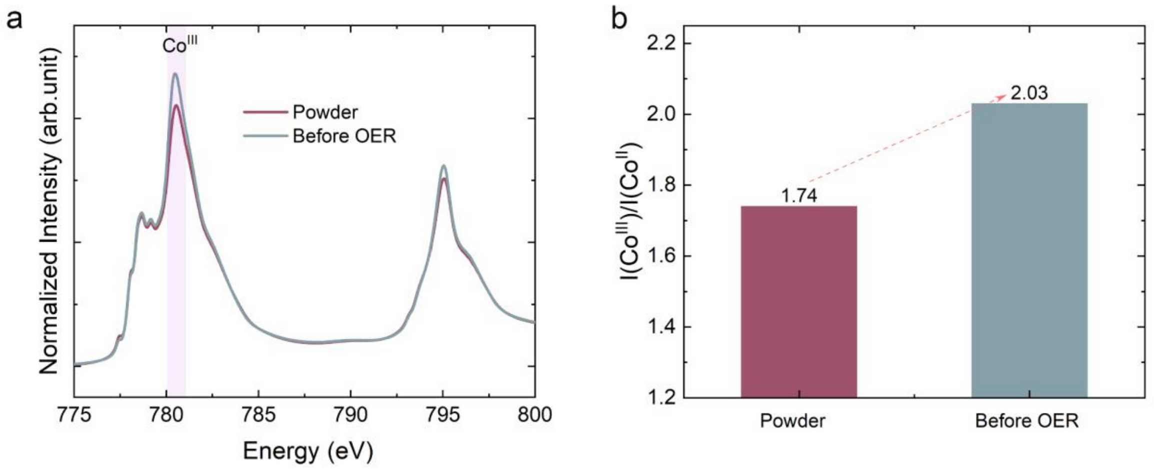

Supplementary Figure 30. (a) Comparison of Co L edge spectra collected for the CoO_x after ink preparation but before any OER tests, with the pristine CoO_x powder. (b) Comparison of the corresponding peak intensity ratio of $I(Co^{III})/I(Co^{II})$ which increases after ink preparation, suggesting the surface species of the pristine CoO_x nanoparticles are already modified upon contacting with water.

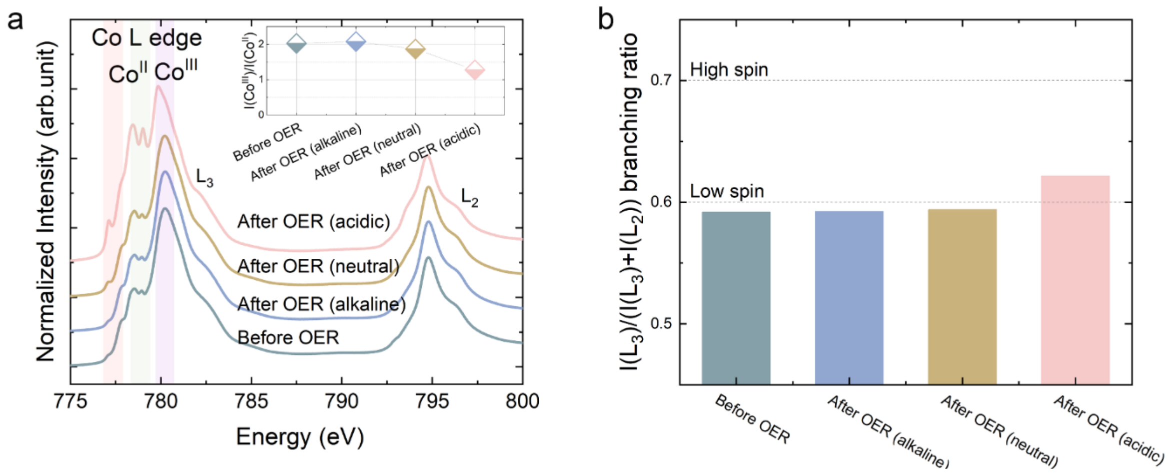

Supplementary Figure 31. Comparison of the surface Co species after OER in different electrolytes. (a) The ex-situ Co L edges and (b) the corresponding calculated $I(L_3)/(I(L_3)+I(L_2))$ branching ratio.

Supplementary Note V. Influence of rotation on the local pH and OER performance in phosphate buffer (pH = 7).

The OER performance in neutral phosphate buffer is significantly affected by the rotation speed of the rotating disk electrode (RDE). The OER current under the same applied potential was decreased when reducing the rotation speed (Supplementary Figure 32a). A similar phenomenon was still observed when the electrolyte was changed to 1 M phosphate buffer (Supplementary Figure 32b). The decrease of performance is more prominent when the rotation is decreased from 1600 rpm to 0 rpm (Supplementary Figure 32c-d).

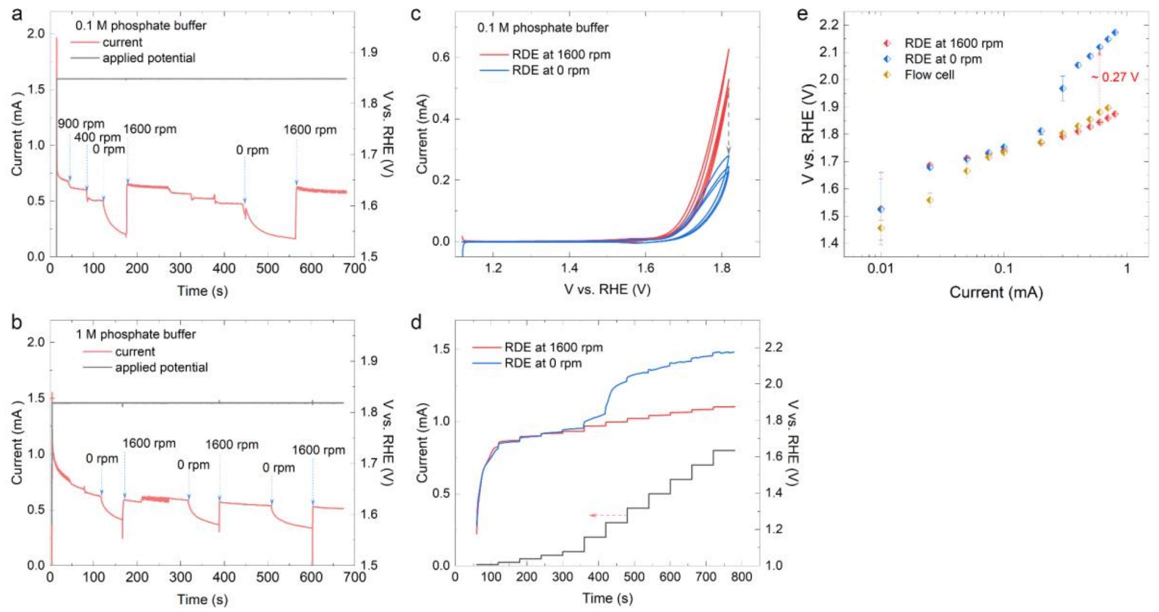

Supplementary Figure 32. Discussing the influence of rotation on the local pH and OER performance in neutral environment using rotating disk electrode (RDE) setup. The i-t curves recorded in (a) 0.1 M phosphate buffer and (b) 1 M phosphate buffer under different rotation speed. Comparison of (c) CV curves and (d) steady state chronoamperometry results at 1600 rpm and 0 rpm. (e) Comparison of the OER performance collected in the RDE setup with that in the flow cell. SD of the applied potential is obtained from average all the potentials recorded at each step (n = 120). Data are presented as mean values +/- SD.

Based on the above observations, it is clear the local pH environment under OER conditions is changed without the rotation. An increase of around ~0.27 V in the applied potential is observed when reducing the rotation from 1600 rpm to 0 rpm (Supplementary Figure 32e), i.e., local pH is reduced by a factor of ~4.5. Therefore, the local pH could be around ~ 2.5 without the rotation, which explains the detection of Co dissolution from ICP-OES (Supplementary Table 5), though the concentration is lower than that in 0.05 M H_2SO_4 (pH ~ 1). It is important to note that at the same OER current, the applied potential in the flow cell (with a flow rate of 4 mL per minute) is slightly higher than in the RDE setup with rotation at 1600 rpm, indicating a slight

change in the local pH environment (but not to the state of the RDE without rotation). As a result, the applied potential measured in 0.1 M phosphate buffer in the flow cell is slightly higher than it should be due to the slight change of local pH. Therefore, we want to note that it is important to rotate the electrode or flow the electrolyte to maintain a homogenous pH environment in a neutral buffer, which is extremely crucial for the operando characterization.

Supplementary Note VI. Discussions on the hXAS data analysis

Due to the bulk sensitivity of hXAS, the signal at the Co K edge is an average of the surface and bulk Co atoms. Therefore, significant changes in the Co oxidation state at the surface may still result in small changes in the detected Co K-edge spectra. To reflect the evolution of Co oxidation state, we have followed strict protocols to analyze the hXAS data.

Data Calibration, Normalization, and Alignment. During the data collection, the Co K edge of both the sample and Co foil were collected simultaneously. Co foil was used as the reference for calibration, with the standard adsorption edge at 7709 eV. We want to note that the spectra of the Co foil collected at different time and during different experiments could be slightly different (Supplementary Figure 33a). To reduce the error from these slight changes, the hXAS spectra of the sample collected during a continuous measurement (e.g., 3 CV cycles or CP measurement) were calibrated using the same Co foil spectra, which is the average result from 60 spectra collected in the first 30 s. During the normalization, the pre-edge range is always set to be -104 to -50 eV (relative to 7709 eV), while the normalized range is 100 to 875 eV. To reduce the error from the calibration, the Co foil reference spectra from different measurements have been further aligned using Athena. As shown in Supplementary Figure 33b, the spectra were aligned according to the maximum of the derivative, which is located at around 7709 eV. It should be noted that, there is uncertainty during this alignment, as also shown in the Supplementary Figure 20d.

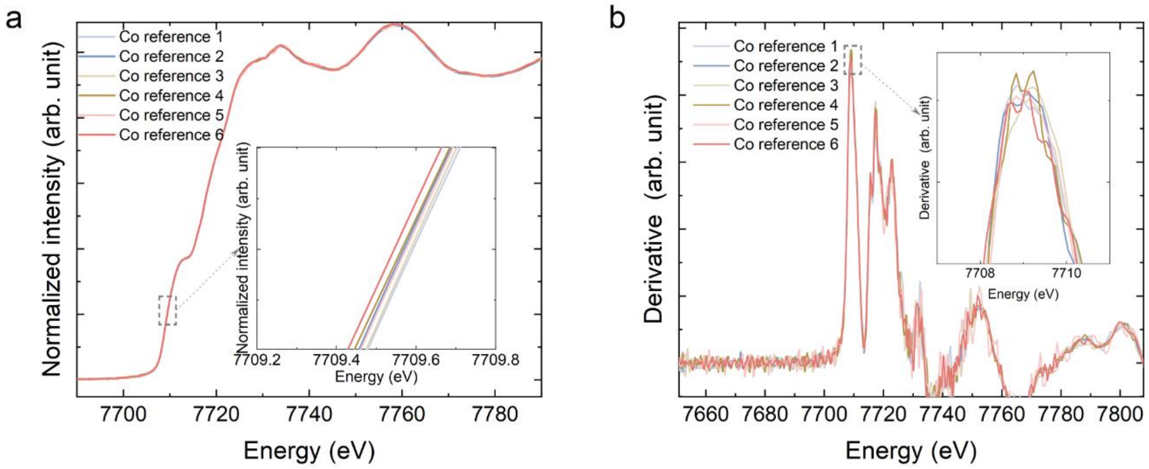

Supplementary Figure 33. (a) The Co foil reference spectra collected simultaneously with Co K edge of the catalyst during the OCP measurement before and after CV in alkaline (Co reference 1 and 2), neutral (Co reference 3 and 4) and acidic (Co reference 4 and 5) electrolytes, respectively. The inset is the enlargement to show the difference at the adsorption edge. (b) The corresponding derivative of the Co foil reference spectra. Inset shows the maximum of the derivative is at ~ 7709 eV.

References in Supplementary Information

1. Huang JZ, Hales N, Clark AH, Yüzbasi NS, Borca CN, Huthwelker T*, et al.* Operando Tracking the Interactions between CoO_x and CeO_2 during Oxygen Evolution Reaction. *Adv. Energy Mater.* 2024, **14**(11)**:** 2303529.

2. Brezesinski T, Wang J, Polleux J, Dunn B, Tolbert SH. Templated nanocrystal-based porous TiO(2) films for next-generation electrochemical capacitors. *J. Am. Chem. Soc.* 2009, **131**(5)**:** 1802-1809.

3. Xiao J, Zhang F, Tang K, Li X, Wang D, Wang Y*, et al.* Rational Design of a P2-Type Spherical Layered Oxide Cathode for High-Performance Sodium-Ion Batteries. *ACS Cent. Sci.* 2019, **5**(12)**:** 1937-1945.

4. Gharbi O, Tran MTT, Tribollet B, Turmine M, Vivier V. Revisiting cyclic voltammetry and electrochemical impedance spectroscopy analysis for capacitance measurements. *Electrochim. Acta* 2020, **343**.

5. Laschuk NO, Easton EB, Zenkina OV. Reducing the resistance for the use of electrochemical impedance spectroscopy analysis in materials chemistry. *RSC Adv.* 2021, **11**(45)**:** 27925-27936.

6. Hankin A, Bedoya-Lora FE, Alexander JC, Regoutz A, Kelsall GH. Flat band potential determination: avoiding the pitfalls. *J. Mater. Chem. A* 2019, **7**(45)**:** 26162-26176.

7. Wang YB, Yuan RK, Willander M. Capacitance of semiconductor-electrolyte junction and its frequency dependence. *Appl. Phys. A* 1996, **63**(5)**:** 481-486.

8. Mefford JT, Akbashev AR, Kang M, Bentley CL, Gent WE, Deng HD*, et al.* Correlative operando microscopy of oxygen evolution electrocatalysts. *Nature* 2021, **593**(7857)**:** 67-73.

9. Deng J, Nellist MR, Stevens MB, Dette C, Wang Y, Boettcher SW. Morphology Dynamics of Single-Layered Ni(OH)2/NiOOH Nanosheets and Subsequent Fe Incorporation Studied by in Situ Electrochemical Atomic Force Microscopy. *Nano Lett.* 2017, **17**(11)**:** 6922-6926.

10. Tang Y, Wu C, Zhang Q, Zhong H, Zou A, Li J*, et al.* Accelerated Surface Reconstruction through Regulating the Solid-Liquid Interface by Oxyanions in Perovskite Electrocatalysts for Enhanced Oxygen Evolution. *Angew. Chem. Int. Ed.* 2023, **62**(37)**:** e202309107.

11. Nong HN, Falling LJ, Bergmann A, Klingenhof M, Tran HP, Spori C*, et al.* Key role of chemistry versus bias in electrocatalytic oxygen evolution. *Nature* 2020, **587**(7834)**:** 408-413.

12. Huang J, Sheng H, Ross RD, Han J, Wang X, Song B*, et al.* Modifying redox properties and local bonding of Co_3O_4 by CeO_2 enhances oxygen evolution catalysis in acid. *Nat. Commun.* 2021, **12**(1)**:** 3036.

13. Hein D, Wartner G, Bergmann A, Bernal M, Roldan Cuenya B, Seidel R. Reversible Water-Induced Phase Changes of Cobalt Oxide Nanoparticles. *ACS Nano* 2020, **14**(11)**:** 15450-15457.

14. Huang J, Borca CN, Huthwelker T, Yuzbasi NS, Baster D, El Kazzi M*, et al.* Surface oxidation/spin state determines oxygen evolution reaction activity of cobalt-based catalysts in acidic environment. *Nat. Commun.* 2024, **15**(1)**:** 3067.

15. Haase FT, Bergmann A, Jones TE, Timoshenko J, Herzog A, Jeon HS*, et al.* Size effects and active state formation of cobalt oxide nanoparticles during the oxygen evolution reaction. *Nat. Energy* 2022, **7**(8)**:** 765-773.

16. Yang J, Liu H, Martens WN, Frost RL. Synthesis and Characterization of Cobalt Hydroxide, Cobalt Oxyhydroxide, and Cobalt Oxide Nanodiscs. *J. Phys. Chem. C* 2010, **114**(1)**:** 111-119.

17. Chong L, Gao G, Wen J, Li H, Xu H, Green Z*, et al.* La- and Mn-doped cobalt spinel oxygen evolution catalyst for proton exchange membrane electrolysis. *Science* 2023, **380**(6645)**:** 609-616.

18. Bloor LG, Molina PI, Symes MD, Cronin L. Low pH Electrolytic Water Splitting Using Earth-Abundant Metastable Catalysts That Self-Assemble in Situ. *J. Am. Chem. Soc.* 2014, **136**(8)**:** 3304-3311.

19. Zhang X, Feng C, Dong B, Liu C, Chai Y. High-Voltage-Enabled Stable Cobalt Species Deposition on MnO_2 for Water Oxidation in Acid. *Adv. Mater.* 2023, **35**(13)**:** e2207066.

20. Priamushko T, Franz E, Logar A, Bijelic L, Guggenberger P, Escalera-Lopez D*, et al.* Be Aware of Transient Dissolution Processes in Co(3)O(4) Acidic Oxygen Evolution Reaction Electrocatalysts. *J. Am. Chem. Soc.* 2025, **147**(4)**:** 3517-3528.
